# Diversity of Brain Microvascular Endothelial Cell Functions: Physiology, Ischemia/Hypoxia, and Underlying Protection

**DOI:** 10.14336/AD.2025.0664

**Published:** 2026-09-17

**Authors:** Mingxuan Cao, Jia Liu, Xunming Ji

**Affiliations:** ^1^Department of Neurology, Xuanwu Hospital, Capital Medical University, Beijing, China.; ^2^Beijing Institute of Brain Disorders, Laboratory of Brain Disorders, Hypoxia Conditioning Translational Laboratory of Clinical Medicine, Ministry of Science and Technology, Collaborative Innovation Center for Brain Disorders, Capital Medical University, Beijing, China.; ^3^Chinese Institutes for Medical Research, Beijing, China

**Keywords:** ischemic stroke, BMECs, functions, physiology, ischemia, hypoxia

## Abstract

Ischemic stroke is a global health crisis associated with high mortality, long-term disability, and high healthcare costs. Microvascular dysfunction is pivotal in its pathophysiology. The brain microvasculature, far from a passive set of structures, is a complex system participating in multiple biological processes, including controlling angiogenesis, maintaining blood-brain barrier (BBB) integrity, regulating cerebral blood flow (CBF) via neurovascular coupling, managing substance transport, and modulating central nervous system immune responses. Brain microvascular endothelial cells (BMECs) are central to the pleiotropic functions of the brain microvasculature; however, ischemia and hypoxia severely affect BMECs and disrupt microvascular functions. Angiogenesis is dysregulated, the BBB becomes permeable, the regulation of CBF becomes unbalanced, nutrient transport is disrupted, and immune modulation is disrupted. Targeted strategies to safeguard BMECs against ischemic-hypoxic injury have great potential for the treatment of ischemic stroke patients. This review comprehensively outlines the diversity of BMECs under normal conditions, delves into the effects of ischemia and hypoxia on these cells, and explores emerging protective strategies. By integrating these elements, this study offers a holistic perspective on the role of BMECs in ischemic stroke pathophysiology and aims to inspire future research in this crucial field.

## 1. Introduction

Ischemic stroke ranks among the most critical global health concerns, as it imposes a substantial burden in terms of mortality, long-term disability, and associated healthcare costs [1, 2]. Accumulating evidence indicates that microvascular dysfunction is at the center of the pathophysiological cascade underlying ischemic cerebro-vascular disease [3]. The brain microvasculature is not a passive set of vessels but a highly sophisticated system that performs diverse and indispensable functions. These functions span the orchestration of angiogenesis during both cerebral repair and pathological conditions [4], the maintenance of blood-brain barrier (BBB) integrity [5], the facilitation of neurovascular coupling for precise cerebral blood flow (CBF) regulation [6], the regulation of bidirectional substance transport between the bloodstream and the brain parenchyma [7], and the modulation of immune responses within the central nervous system (CNS) [8, 9]. Brain microvascular endothelial cells (BMECs) constitute the cornerstone of the microvascular system and are the primary determinants of its functional diversity [10]. Therefore, a better understanding of BMEC functions under normal and pathological conditions is highly important for elucidating the pathogenesis of ischemic stroke and for developing novel therapeutic strategies.

Ischemia and hypoxia, the hallmarks of ischemic stroke, initiate a series of deleterious events that primarily target BMECs. These processes lead to a cascade of functional impairments in the microvasculature. For example, angiogenic processes are either aberrantly activated or inhibited, impeding the restoration of the blood supply to the ischemic region [11]. The BBB becomes compromised as tight junctions (TJs) between BMECs are disrupted, which results in increased permeability and the infiltration of harmful substances into the brain [12]. Impaired neurovascular coupling disrupts CBF regulation [13]. Nutrient and metabolite transport across BMECs is dysregulated, and while neurons are deprived of essential substrates, they may also accumulate neurotoxic molecules [14]. The immunoregulatory functions of BMECs are also altered, often leading to an exacerbated inflammatory response in the CNS [15]. Given the central role of BMECs in maintaining microvascular integrity and function, the development of targeted strategies to protect BMECs from ischemic-hypoxic injury holds great promise as a novel therapeutic approach for ischemic stroke. Such strategies could preserve the functional integrity of microvasculature to mitigate the severity of brain damage and promote better clinical outcomes.

In this review, we systematically summarize the current understanding of the diversity of BMECs under physiological conditions. We then delve into the profound impacts of ischemia and hypoxia on BMECs and highlight how these insults lead to the disruption of microvascular functional diversity. Furthermore, we explore emerging strategies that aim to target and protect BMECs, providing insights into potential therapeutic interventions for ischemic-hypoxic brain diseases. By integrating these aspects, we seek to provide a holistic view of the role of BMECs in ischemic stroke pathophysiology and to inspire future research in this critical area.

## 2. Diversity of BMECs under physiological conditions

Endothelial cells (ECs) are not just inert cells that form the blood vessel lining; they also actively participate in angiogenesis, both in health and disease [16]. In the brain, BMECs are involved in the function of the BBB as physical barriers, transport barriers, and immune barriers [17]. With the introduction of the neurovascular unit (NVU), the role of BMECs in sensing neuronal activity and regulating CBF has gradually been identified [18]. Recently, several studies have investigated the roles of shear stress (SS) [19, 20] and angiophagy [21, 22] in BMECs and have increased our understanding of the diversity of BMECs. Therefore, we first review the functional diversity of BMECs under physiological conditions (Fig. 1).

### 2.1 Role of BMECs in angiogenesis

Angiogenesis, the formation of new blood vessels, is a dynamic process during which ECs proliferate and migrate, resulting in vascular network expansion and remodeling [23]. Brain ECs originate in the perineural vascular plexus surrounding the developing neural tube [24]. Five EC subtypes, including mitotic, venous, capillary, tip, and arterial ECs, are present in the prenatal human brain at 15-23 gestational weeks. RNA sequencing of the EC transcriptome revealed a developmental progression from immature venous and mitotic ECs through capillary/tip cells to arterial ECs [25], as tip and mitotic cells are transient ECs [23]. In the adult mouse brain, only three distinct EC subtypes remain, namely, the venous, capillary, and arterial subtypes [25]. The primary mechanisms regulating angiogenesis during development involve vascular endothelial growth factor-A (VEGF-A), its receptors (VEGFRs) and Notch/Delta-like Notch ligands [26]. During angiogenesis, some ECs within the capillary wall are selected for sprouting. ECs differentiate into tip or stalk cell phenotypes. Filopodia on tip cells guide sprout elongation toward the angiogenic stimulus. Delta-like-4 (DLL4) is expressed in tip cells, and the activation of Notch signaling in neighboring ECs is thought to inhibit the sprouting of these cells. Decreased levels of DLL4 or blockade of Notch signaling increase tip cell formation and endothelial tube sprouting, branching, and fusion, resulting in endothelial sprouts that fail to prevent excessive angiogenic motility and branching [27]. Stalk cells exhibit high proliferation and elevated Notch1 expression, which supports new sprout extension [26]. In addition, the formation of tubules by ECs is another critical step in angiogenesis and requires coordinated cytoskeletal and cell-matrix adhesion functions [11].

Like any other organ, angiogenesis is regulated by the coordinated balance of proangiogenic and antiangiogenic factors [24]. For example, pituitary adenylate cyclase-activating polypeptide (PACAP), an evolutionary conserved neuropeptide, possesses significant antiaging properties. PACAP promotes capillary-like structure formation by ECs, and the dysregulation of autocrine PACAP signaling due to aging contributes to the impaired angiogenic capacity of BMECs [28]. Autophagy-related gene 7 (Atg7) is an autophagy-related protein homologous to ubiquitin-activating enzyme E1 that promotes BMEC tube formation. On the one hand, Atg7 can regulate the transcription of interleukin-6, an important proangiogenic factor involved in angiogenesis, through the nuclear translocation of NF-κB [29]. On the other hand, Atg7 promotes tube formation by modulating laminin-5 expression through NF-κB [30]. Hydrogen peroxide (H_2_O_2_) is an important endogenous reactive oxygen species (ROS), and H_2_O_2_ can induce angiogenesis during embryonic development and postsurgical wound healing. Similarly, in BMECs, low concentrations of H_2_O_2_ (0.001-1 µM) promote an increase in the total tube length [31]. Nogo-A functions not only as an axonal growth inhibitor but also as a negative regulator of angiogenesis during development. *In vitro* experiments confirmed that Nogo-A induces the retraction of BMEC lamellipodia and filopodia while inhibiting their migration, spreading, and sprouting. Similarly, the knockdown of Nogo-A *in vivo* promotes a significant increase in the vascular density [32].

**Figure 1.**
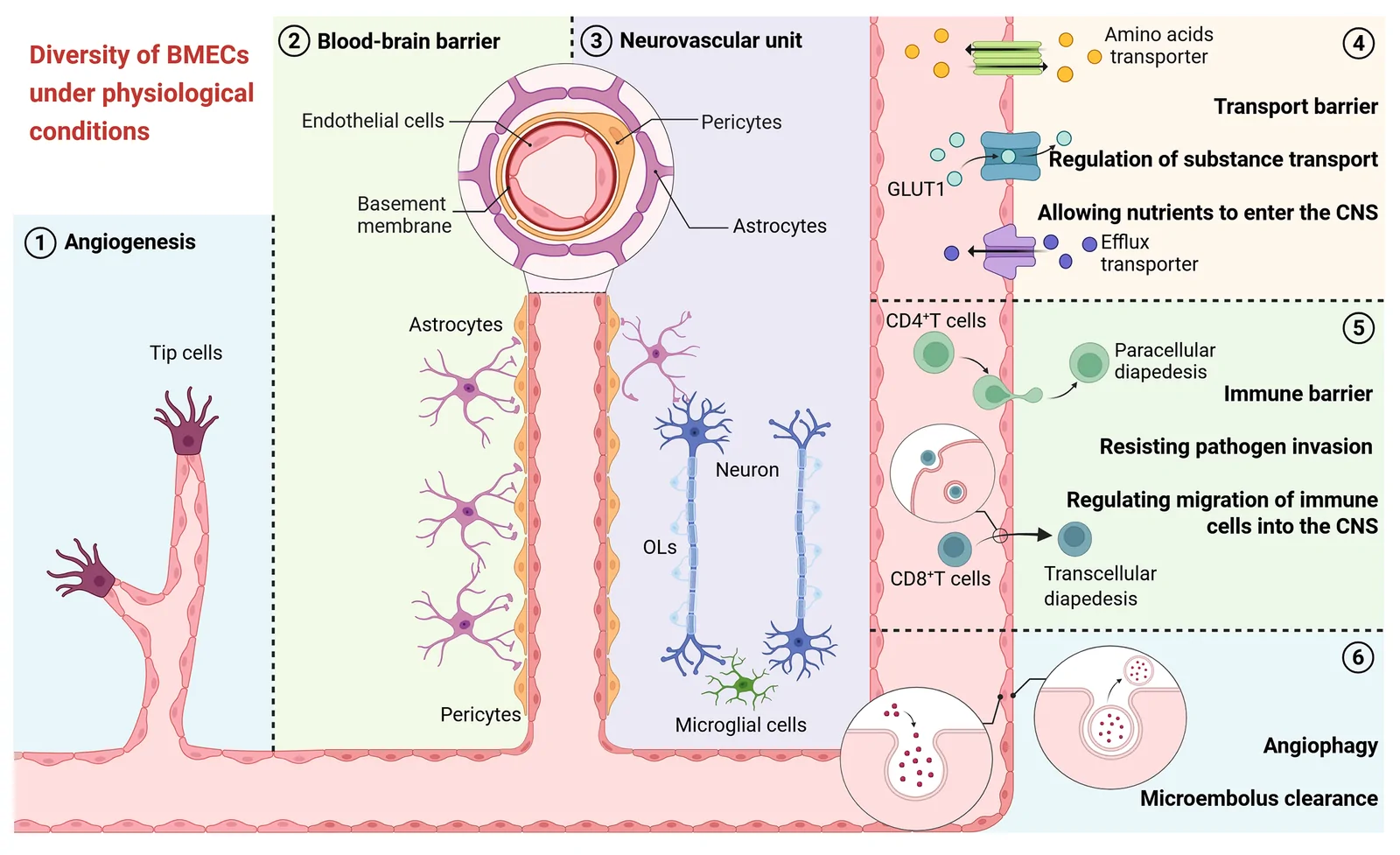
**Diversity of BMECs under physiological conditions**. BMECs have several critical biological functions, including ① engaging in angiogenesis; ② participating in the formation of the blood-brain barrier; ③ participating in the formation of the neurovascular unit for cerebral blood flow regulation; ④ forming a transport barrier and regulating substance transport; ⑤ acting as an immune barrier against pathogen invasion, which regulates the migration of immune cells into the CNS; and ⑥ and mediating other functions, such as angiophagy. *BMECs, brain microvascular endothelial cells; CNS, central nervous system; GLUT1, glucose transporter 1; OLs, oligodendrocytes.

### 2.2 The maintenance of BBB integrity by BMECs

The BBB, which is composed of BMECs, pericytes, the basement membrane, and the endfeet of astrocytes, is a physical and biological barrier between cerebral blood vessels and brain tissue that maintains the cerebral microenvironment [33]. Compared with other ECs, which are important components of the BBB, BMECs present characteristics such as high-resistance TJs, control of metabolite transcellular transport, restriction of paracellular transport, an absence of fenestrations (small pores enabling rapid molecular passage), and extremely low transcytosis rates [34]. The expression of TJs and the markedly asymmetric distribution of transmembrane transporter proteins contribute to BMEC polarization [35]. These features allow the BBB to inhibit the uncontrolled diffusion of water-soluble molecules and prevent neurotoxic protein entry into the brain through the enrichment of polarized efflux transporters; however, the BBB still allows the diffusion of nutrients, which is assisted by distinct nutrient transporters [17]. In general, the functionality of the BBB relies primarily on two barriers: the physical barrier formed by TJs and the transport barrier formed by transporters and vesicles. The physical barrier is described here, whereas the transporter barrier is further described in a later section.

The combined actions of TJs and adherens junctions (AJs) restrict BBB intercellular gaps to approximately 1.4-1.8 nm, ensuring strictly controlled paracellular transport [36]. TJ proteins include occludins (OCLNs), claudins (CLDNs), junctional adhesion molecules (JAMs), and their adaptor protein zonula occludens-1 (ZO-1), which are central structures that mediate BBB impermeability and low cellular paracellular flux [17]. CLDN5 is the most abundant TJ protein expressed in the BMECs. CLDN5 exhibits size-selective properties against small molecules (less than 800 Da) [37]. Interestingly, BMECs do not spontaneously express high levels of TJ proteins, as TJ protein expression is induced by surrounding perivascular cells, pericytes, and astrocytes, among which astrocytes play a pivotal role [38]. The major components of AJs include vascular endothelial (VE)-cadherin, neural-cadherin, and platelet endothelial cell adhesion molecule-1 (PECAM-1) [39]. VE-cadherin couples with the actin cytoskeleton, thereby providing a signaling hub for barrier regulation [24]. Under normoxic conditions, parallel F-actin bundles are observed in BMECs. These bundles localize either at cell-cell boundaries or form the cortical actin rim—a key element for endothelial tightness [40]. In addition to TJs (which regulate paracellular solute passage) and AJs (which stabilize intercellular adhesion), maintaining BBB integrity and permeability also requires gap junctions [41]. Gap junctions are intercellular channels connected to the cytoplasm of an adjacent cell. Unlike TJs and AJs, gap junctions lack tight seals, permitting ion and small molecule (<1.5 kDa) exchange between neighboring cells [42]. These well-structured junctional complexes confer high physiological transepithelial electrical resistance values (>1000 Ω/cm^2^) to the brain capillaries, which are much higher than those of other tissues (3-33 Ω/cm^2^) [43].

One of the crucial determinants that regulate BBB permeability is the modulation of endothelial intercellular junctions. Factors such as the cellular prion protein, a key surface adhesion molecule, increase the integrity and functional barrier properties of the BBB by increasing TJ protein expression [44]. Additionally, insulin increases the integrity of TJs in human BMECs (HBMECs) through the PI3/AKT/GSK-3β signaling pathway [45]. Research has focused on the Rho GTPase (including RhoA and Rac1) signaling pathway, which is pivotal for maintaining BBB homeostasis. Assembly and disassembly of endothelial cytoskeletal proteins, mediated by the Rho GTPase signaling pathway, represent the primary mechanism regulating intercellular junction homeostasis and, consequently, BBB permeability. For instance, the activation of the RhoA/ROCK/pMLC pathway by proinflammatory cytokines or growth factors promotes stress fiber formation and junction disruption, increasing paracellular permeability [8]. In contrast, inhibiting poly (ADP-ribose) polymerase-1 alleviates BBB damage by inhibiting RhoA/Rac1 activity and increasing TJ protein expression in the brain endothelium [46]. However, more facilitators are not necessarily better, and the concentration is gradually being scrutinized. For example, retinoic acid (RA), an essential morphogen that regulates embryonic development, upregulates TJs, but its homeostasis is regulated by P450 oxidoreductase (POR). POR deficiency causes pathological RA accumulation, which impairs RA homeostasis, promotes inflammatory responses, and consequently disrupts BBB integrity [47].

### 2.3 Support of the NVU structure by BMECs

Research on the BBB has revealed close connections and interdependence between the vasculature and the neuronal network [6]. Anatomically, a consistent parallel relationship has been observed between nerves and blood vessels, and each neuron is located less than 15 µm from a capillary to ensure perfusion throughout the brain [48]. During CNS development, neurons and oligodendrocyte precursor cells migrate along blood vessels. Neurovascular interactions established during development continue to function in adulthood to coordinate the maintenance of CNS homeostasis. Owing to its high energy and oxygen requirements and lack of energy reserves, the brain relies on sustained perfusion, which requires unique, dynamic systems for cerebral hemodynamic control [49]. The NVU provides a useful framework for studying how neuronal signaling modulates the nearby microvasculature to meet cerebral metabolic demands. The NVU consists of vascular cells (BMECs, pericytes, and smooth muscle cells), neurons, glial cells (oligodendrocytes, microglia, and astrocytes), and the basal lamina matrix [37]. The mechanism of the NVU is believed to be initiated by the release of glutamic acid from activated neurons, which then activates astrocytes, pericytes, and neighboring neurons, triggering the secretion of vasoactive mediators. This resulting balance between vasoconstriction and vasodilation modulates local CBF [50].

BMECs coordinate neurovascular functions through dynamic communication with neighboring NVU cells. In the regulation of CBF, cerebral capillaries act as neuronal activity-sensing networks that initiate hyperpolarizing signals to regulate local CBF [18]. For example, neuronal activity stimulates the transient receptor potential ankyrin 1 (TRPA1) channel in BMECs, triggering signals that induce microarteriolar dilation to initiate functional hyperemia. Functional hyperemia can not only ensure the energy consumption of different brain regions in the resting state but also ensure a rapid increase in CBF in the corresponding brain region when neuronal activity increases; namely, blood is redirected to regions of metabolic demand [51]. In addition to TRPA1 channels, BMECs also sense neuronal activity via N-methyl-D-aspartate receptors and inward-rectifying K^+^ (Kir2.1) channels [52]. Pericytes and astrocytes also play important roles in this process. The pericyte processes encircle capillaries, covering approximately 90% of their surface. As candidates for regulating microcirculatory blood flow, pericytes are strategically located along microvessels and exhibit rapid responsiveness to neuronal stimuli [53]. Astrocytes not only monitor neuronal activity and respond to neuronal metabolic changes but also regulate pericyte/smooth muscle cell contraction and relaxation through the secretion of vasoactive substances, thereby instantaneously regulating CBF according to neuronal activity [54]. Astrocytes are also considered essential supporters of BMECs. Many factors from astrocytes are involved in regulating BMECs. For example, astrocyte-derived angiotensin acts on angiotensin receptors in BMECs to maintain low infiltration of the BBB. Astrocyte-derived transforming growth factor (TGF)-β1 increases ZO-1 expression in BMECs through a noncanonical hedgehog signaling pathway [55]. *In vitro* studies revealed the effects of coculture of astrocytes and pericytes on HBMECs and showed that ZO-1 expression and localization at cell-cell junctions increased in this coculture system [56].

### 2.4 Mechanisms involved in substance transport in BMECs

Under normal physiological conditions, BMECs secrete Na^+^, Cl^-^, and water into the brain parenchyma while extruding K^+^ to maintain physiological extracellular K^+^ concentrations [35]. The BBB not only strictly regulates the chemical composition but also expresses many specific transport proteins and enzymes to ensure that essential nutrients enter the CNS. It simultaneously blocks neurotoxic blood components, pathogens, and potentially toxic metabolites from accessing the brain, thereby maintaining the homeostasis of the cerebral microenvironment and neuronal function [8, 17].

Amino acids (AAs) can be transported in both directions across the BBB. Brain ECs transfer physiological AAs from interstitial fluid into circulation via abluminally localized Na^+^-coupled transporters. Concurrently, luminal membrane transporters for glutamine and glutamate facilitate the clearance of neuroactive AAs from the CNS, maintaining their low cerebral concentrations. This bidirectional and highly regulated mechanism maintains cerebral AA homeostasis [57]. Glucose traverses the BBB predominantly via glucose transporters (GLUTs). GLUT1 is widely considered the primary GLUT [36]. High GLUT1 expression allows glucose to be transported to the brain to meet its energy needs [58]. Fatty acid and transferrin transport depend on transcytosis in BMECs [34]. Docosahexaenoic acid (DHA), a polyunsaturated fatty acid, interacts with the intracellular carrier fatty acid-binding protein 5, completing DHA uptake through endocytosis in BMECs [59]. Iron is an important cofactor involved in neurotransmitter synthesis, myelination, and energy metabolism, which are critical for brain function [60]. During iron transport, transferrin receptors (TfRs) on the surface of BMECs bind to transferrin molecules that carry iron and internalize the receptor-transferrin complex by endocytosis from the luminal (blood-facing) side [61]. On the abluminal (brain-facing) side of BMECs, iron is released from transferrin; thus, it is available for binding to surrounding neurons and glial cells [62]. Recent studies have shown that BMECs not only serve as conduits for iron transport but also work with astrocytes to regulate iron homeostasis in the brain. Astrocyte-generated paracrine signals mediate brain iron delivery to BMECs, and the endosomal cation/proton exchanger NHE9 responds to paracrine signals by increasing the endosomal pH and upregulating TfR, which regulates TfR-dependent iron uptake in BMECs [60]. In contrast, when high intracellular iron levels are present, astrocytes physically contact BMECs and increase hepcidin secretion at their endfeet, which reduces ferroportin 1 expression in BMECs and the uptake of iron from the circulation [63]. Efflux transporters, including P-glycoprotein (P-gp), breast cancer resistance protein, and multidrug resistance-associated proteins, can efflux potentially harmful substrates back into the bloodstream, thereby blocking the CNS penetration of neurotoxic compounds [34].

### 2.5 The immunoregulatory function of BMECs in the CNS

In the BBB, BMECs are the primary cells that initiate the immune response and constitute the first physical and immune barrier that detects and defends against pathogen attack. BMECs are the first-line cells that sense viruses, and when viruses invade the CNS through the BBB, BMECs can rapidly develop an immune response and interact with other cells to interfere with the neural invasion of viruses [8]. Transient receptor potential (TRP) channels expressed on BMECs are members of the cationic cell membrane channel protein superfamily. During the initial stages of the inflammatory process, TRP channels regulate cytokine production and leukocyte adhesion, thereby protecting the CNS from pathogen invasion [64]. BMECs also express classic pattern recognition receptors and specifically express MFSD2a receptors. These receptors are essential for viral recognition and immune responses [8]. In addition, BMECs exhibit autonomous antiviral immunity. For example, induced pluripotent stem cell-derived HBMECs stably express interferon (IFN)-induced transmembrane protein-1, which inhibits the replication of nonneurotropic flaviviruses [58]. In addition, double-stranded DNA mimic poly(dA:dT)-activated HBMECs suppress HSV-1 replication, whereas the double-stranded RNA analog polyI:C initiates the Toll-like receptor (TLR)-3/IFN antiviral pathway in HBMECs [65].

The BBB acts as a bridge between the CNS and immune system, closely controlling immune cell entry into the CNS [66]. In the physiological state, circulating immune cells in the CNS are extremely scarce and restricted to CD4^+^/CD8^+^ T cells, which maintain CNS immunosurveillance [9]. Conversely, neuroinflammatory conditions activate microglia and astrocytes, resulting in the release of proinflammatory cytokines. This secondary disruption of the BBB facilitates substantial immune cell infiltration into the CNS [67]. T cells cross the BBB in two ways. During immunosurveillance processes (homeostasis), T cells polarize and crawl along blood flow to BMECs in an intercellular adhesion molecule (ICAM)-1/ICAM-2-dependent manner. T cells ultimately preferentially cross the BBB via paracellular diapedesis; i.e., they cross between BMEC junctions. Under low inflammatory conditions, low ICAM-1 expression on BMECs directs T cells toward the paracellular route. During exacerbated inflammation, however, elevated ICAM-1 expression shifts the transmigration pathway toward transcellular diapedesis [17]. In inflammatory states, before undergoing diapedesis, encephalitogenic CD4^+^ T cells extend their crawling along blood flow, which is mediated by α4β1 integrin, to search for sites of diapedesis. However, when CD4^+^ T cells polarize and crawl before infiltration, most CD8^+^ T cells undergo transient arrest and preferentially cross the BBB via transcellular routes [66]. PECAM-1 is an important molecule that regulates T-cell migration. When endothelial PECAM-1 is absent, encephalitogenic T cells prefer transcellular diapedesis to paracellular diapedesis to cross the BBB [68]. Additionally, atypical chemokine receptor 1 (ACKR1) promotes transcellular T-cell diapedesis. Under physiological conditions, ACKR1 deficiency reduces this transmigration process [69]. In addition, in the inflammatory state, stationary neutrophils exclusively access paracellular routes to cross the BBB. In contrast, migrating neutrophils primarily traverse endothelial junctions but may also engage in transcellular pathways [9].

### 2.6 Other characteristics and functions of BMECs

SS, the tangential mechanical force exerted by blood flow on cerebral microvascular walls, manifests as laminar flow within brain microvessels. Typical SS values range from 5 to 23 dyn/cm^-2^. BMECs detect this biomechanical stimulus, activating downstream biochemical signaling cascades. The presence of SS induces BMECs to align perpendicular to the direction of blood flow [19]. SS also increases junctional tightness. Physiological shear upregulates the expression of the TJ markers ZO-1 (1.7-fold) and CLDN5 (more than 2-fold) [20]. In addition, SS can activate antioxidant and anti-inflammatory pathways, thereby exerting protective effects on ECs [19].

Effective extravasation mechanisms in the brain remove microemboli from the vasculature [70]. In one *in vitro* study, polystyrene microspheres and fibrin clots were applied to simulate microemboli, and the results revealed that the microspheres were absorbed through endothelial apical membrane cups, while fibrin clots underwent complete cellular engulfment, and no intracellular degradation of the microspheres or fibrin clots was observed. *In vivo* experiments in rats showed that microspheres are transported from microvessels to the brain parenchyma via BMECs [21]. Similarly, *in vivo* imaging in mice revealed that microemboli from different sources are translocated to the perivascular parenchyma through the phagocytosis of the emboli by BMECs, which allows the extravasation of the emboli into the parenchyma, leading to revascularization and the re-establishment of blood flow [22]. This process, termed angiophagy, involves BMECs engulfing circulating microparticles and abluminally extruding them. Angiophagy may act as a protective mechanism that facilitates microvascular recanalization to restore CBF [21].

## 3. Changes in the diversity of BMECs under ischemic/hypoxic conditions

While ischemia and hypoxia cause vascular damage, self-repair is initiated, and angiogenesis is important for injury repair [11]. We therefore realized that the function of BMECs under ischemic/hypoxic conditions is not completely disrupted. On the one hand, ischemia/hypoxia-induced injury to BMECs disrupts BBB function, resulting in dysfunction of transporters and the emergence of an inflammatory response [12]. On the other hand, the angiogenesis of BMECs and the protective effects of other cells in the NVU on BMECs mediate a potential self-rescue mechanism of the vasculature [11].

### 3.1 The involvement of BMECs in ischemia/hypoxia-induced angiogenesis

Angiogenesis is important for the repair of tissue damage and remodeling of the BBB after ischemia to meet the metabolic needs of tissues in the absence of oxygen and nutrients [11]. An increased microvessel density around areas of cerebral infarction is associated with prolonged survival in ischemic stroke patients and improves the patient prognosis [71]. VEGF is a peptide produced by target tissues under hypoxic conditions that activates receptors on BMECs, promotes BMEC proliferation, and is directly involved in angiogenesis in ischemic tissues. This process is governed by hypoxia-inducible factor (HIF)-1α, an intracellular oxygen sensor that ensures spatiotemporally precise angiogenesis where physiologically required [72]. X-box binding protein l splicing (XBP1s) serves as a transcription factor that governs BMEC proliferation and angiogenesis. Under oxygen-glucose deprivation (OGD) conditions, XBP1s overexpression in BMECs reduces cleaved caspase-3/9 levels while activating pro-survival and angiogenic signaling pathways [73]. RNA sequencing technology was used to analyze long noncoding RNAs (lncRNAs) in mouse BMECs (bEnd.3) after OGD or OGD/reoxygenation (OGD/R). Notably, the expression of the G protein-coupled receptor 137b pseudogene (Gpr137b-ps), which functions as a master regulator of angiogenesis, was significantly upregulated [74]. In contrast, the lncRNA myocardial infarction-associated transcript (MIAT) promotes BMEC injury after cerebral ischemia, which is mediated by increased expression of high mobility group box-1 (HMGB1) [75]. HMGB1, a damage-associated molecular pattern, contributes to the pathogenesis of multiple CNS disorders [76]. MIAT inhibition reduces HMGB1 expression, attenuates hypoxia-induced BMEC injury, promotes BMEC angiogenesis, and increases neuronal survival [75]. MicroRNA (miR)-103a also plays a role in BMEC injury in rats with middle cerebral artery occlusion (MCAO). Inhibiting miR-103a upregulates Bcl-2, VEGF, and Ang-1 while downregulating caspase-3 and Bax, collectively suppressing apoptosis and restoring angiogenesis in BMECs [77]. Notably, while angiogenesis serves as a self-repair mechanism and a therapeutic target, increased angiogenesis is not necessarily better—the dose is a critical factor. For instance, in individuals with Moyamoya disease, abnormal angiogenesis does not represent a healthy compensatory outcome. Molecules that promote angiogenesis often increase the proliferation and tube formation of BMECs; however, the unstable state of BMECs during this process also leads to increased vascular permeability. Recent studies have highlighted molecules such as VEGFB that can suppress excessive angiogenesis [78]. Therefore, the balance between promoting angiogenesis and inhibiting its overactivation deserves greater attention.

### 3.2 BMEC injury leads to BBB dysfunction

When ischemia/hypoxia occurs, endothelial damage induces BBB dysfunction, which leads to inflammatory cell infiltration into the cerebral parenchyma [12]. Cytokine-driven inflammation exacerbates BBB disruption, potentiating barrier permeability [79]. Studies have shown that after ischemia/reperfusion (I/R) injury, HBMECs exhibit increased expression of HIF-1α, C-X-C motif chemokine ligand 6, and C-X-C motif chemokine receptor 2 and decreased expression of sirtuin 3 (SIRT3), which inhibits HBMEC proliferation, promotes apoptosis, and in turn promotes BBB disruption [80]. Hypoxia induces increased matrix metalloproteinase (MMP)-9 activity and a disruption of TJ proteins in BMECs, one of the mechanisms that mediates the disruption of BBB function [81]. SIRT5 was shown to mediate BBB damage through the degradation of TJ proteins. SIRT5 silencing upregulates OCLN and CLDN5 expression and decreases BMEC permeability [82]. miR-212/132 is a regulator of the hypoxic BBB. Hypoxia induces miR-212/132 expression in BMECs, resulting in decreased expression of CLDN1, JAM3, and TJ-associated protein 1, which mediate impaired BBB function [83]. Cytoskeleton and cell motility-related proteins also contribute to maintaining the endothelial barrier. Hypoxia leads to F-actin catabolism, which disrupts endothelial integrity [40]. Hypoxia-induced barrier disruption is partially attributed to enhanced actomyosin contractility and fibronectin fibrillogenesis. Under normal conditions, the maintenance of barrier homeostasis relies on appropriate actomyosin contractility. When hypoxia occurs, actomyosin contractility increases above the normal levels, leading to the disassembly of TJs and disruption of the expression of the TJ protein CLDN5. Moreover, hypoxia stimulates fibronectin fibrillogenesis, which further increases contractility and disrupts cell-cell junctions [7]. Nonmuscle myosin II is a motor protein with light and heavy chains that regulate its contractile properties. Nonmuscle myosin heavy chain IIA (NMMHC IIA) is highly expressed in BMECs with I/R injury, where it disrupts the integrity of the BBB. The knockdown or inhibition of NMMHC IIA expression reverses BBB damage by inhibiting TJ degradation [84]. The tightness of intercellular junctions, especially adhesive junctions consisting of homotypic cell-cell adhesion mediated by VE-cadherin, is a key determinant of vascular permeability. An *in vitro* cellular assay revealed that OGD/R increases the permeability of HBMEC monolayers by triggering VE-cadherin endocytosis via the RhoA/ROCK2 pathway [85]. Therefore, the destabilization of VE-cadherin is also an important mechanism of hypoxia-induced BBB damage. Class II phosphoinositide 3-kinase β inactivation maintains the stability of the VE-cadherin protein at adhesion junctions, thereby protecting endothelial barrier integrity [79]. Glycocalyx (GCX) is a key structure that regulates BBB integrity. Following I/R, GCX exhibits dual-phase degradation-reconstitution-degradation dynamics that parallel biphasic alterations in BBB permeability. GCX degradation coincides with increased endothelial transcytosis, consequently increasing barrier permeability. Knockdown of caveolin-1 expression suppresses endothelial transcytosis and leads to decreased BBB permeability and brain edema. Thus, GCX degradation compromises the BBB through caveolae-mediated transcytosis mechanisms [86]. In summary, the core mechanisms by which ischemia/hypoxia damage BMECs include the following: first, they directly target and degrade TJ and AJ proteins; second, they induce cytoskeletal contraction, mechanically disrupting intercellular connections; and third, the protective GCX molecule on the cell surface is damaged, leading to uncontrolled transcytosis. These intricate pathways ultimately converge on a common outcome: increased BBB permeability. We summarize the mechanisms leading to increased BBB permeability in Figure 2.

**Figure 2.**
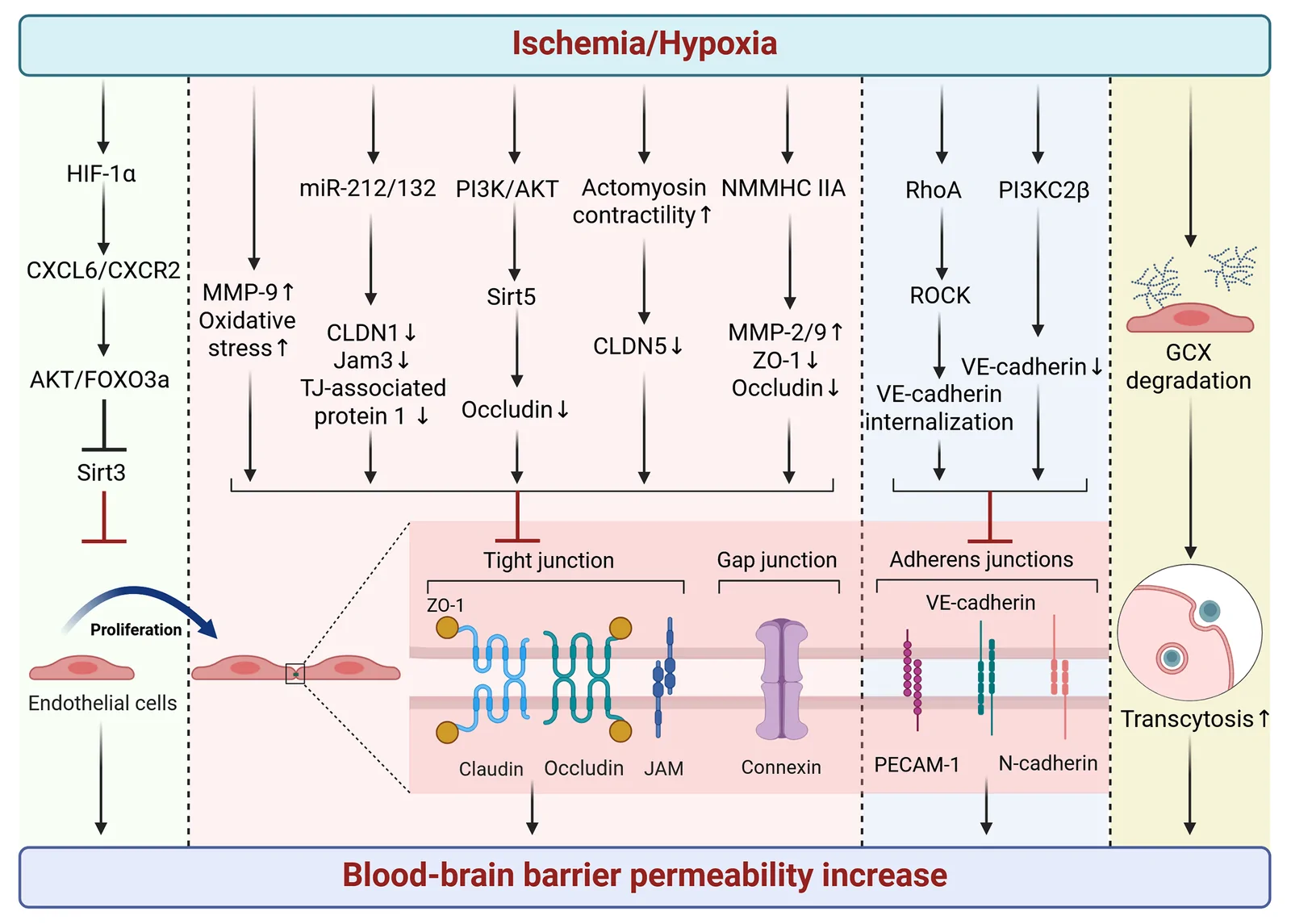
**The mechanism of increased BBB permeability under ischemic/hypoxic conditions**. Under ischemic and hypoxic conditions, the mechanisms that mediate increased BBB permeability include ① the inhibition of endothelial cell proliferation; ② the destruction of TJs; ③ increased actomyosin contractility; ④ the destabilization of VE-cadherin and the disruption of adherens junctions; and ⑤ the degradation of the glycocalyx along with an increase in endothelial transcytosis. *BBB, blood-brain barrier; HIF, hypoxia inducible factor; CXCL, C-X-C motif chemokine ligand; CXCR, C-X-C motif chemokine receptor; MMP, matrix metalloproteinase; miR, microRNA; CLDN, claudin; TJ, tight junction; NMMHC IIA, nonmuscle myosin heavy chain IIA; ZO-1, zonula occludens-1; JAM, junctional adhesion molecule; VE, vascular endothelial; PI3KC2β, class II phosphoinositide 3-kinase β; GCX, glycocalyx.

BMEC damage represents a prominent pathological hallmark of BBB injury; therefore, BMEC damage is a significant therapeutic target in ischemic stroke. Basic fibroblast growth factor (bFGF) not only mediates angiogenesis and neurogenesis but also protects against BBB disruption. *In vitro* cellular experiments revealed that bFGF significantly reduces OGD/R-induced BMEC apoptosis and inhibits the downregulation of TJs and AJs through the activation of the FGF receptor 1 and ERK pathways, thereby decreasing BBB permeability [87]. Under hypoxic conditions, the expression of the lncRNA MACC1-AS1 is reduced in HBMECs. This molecule exerts endothelial-protective effects through the miR-6867-5p/TWIST1 axis, whereby its overexpression attenuates apoptosis and oxidative stress while increasing VE-cadherin expression with concomitant barrier preservation through decreased permeability [88]. A recent study revealed two brain EC-specific genes, AURKA and CENPF, and RNA regulatory axes (miR297b-3p-CENPF and miR34b-3p-CENPF) as potential therapeutic targets for ischemic stroke [89]. A-kinase anchor protein 12 (AKAP12), a scaffolding protein, attenuates ischemic stress-induced BBB damage and dysfunction. AKAP12 depletion activates the Rho kinase pathway, contributing to increased endothelial permeability [90]. Oxidative stress constitutes a key pathogenic mechanism underlying cerebral ischemia-induced BBB damage, and antioxidants can inhibit ROS production, thereby attenuating BBB damage. Peroxiredoxin 4, a highly effective H_2_O_2_ scavenger, is directly involved in protecting BMECs from ROS and acts as a membrane-associated peroxidase in BMECs [91]. BMEC autophagy is also critical for the protection against and the repair of vascular injury induced by cerebral ischemia. Ischemic/hypoxic stress induces the redistribution of the TJ protein CLDN5 from plasma membranes to cytoplasmic compartments in BMECs, increasing BBB permeability. Autophagy inhibits the CLDN5 redistribution and removes abnormally accumulated CLDN5 from the cytoplasm, thereby maintaining BBB integrity [92]. The lncRNA MALAT1, whose expression is significantly upregulated in BMECs under ischemic conditions, modulates cellular autophagy to mitigate ischemic brain injury. It downregulates both miR-26b [93] and miR-200c-3p [94], thereby counteracting their inhibitory effects on BMEC autophagy. However, decreased autophagy is not always reflected in the negative effects on BMECs. For example, the knockdown of the important autophagy regulator autophagy-related 3 inhibits autophagy, which ameliorates OGD/R-induced inflammation and injury in BMECs through the PI3K/AKT pathway [95]. In summary, current therapeutic strategies targeting BMEC injury in ischemic stroke focus primarily on restoring and maintaining BBB integrity. These interventions include both the exogenous administration of protective factors and endogenous gene regulation strategies. Key mechanisms involve stabilizing intercellular junctions, inhibiting apoptosis, reducing oxidative stress, and modulating autophagy.

### 3.3 The role of BMECs in NVU repair in ischemic brain injury

In recent years, an increasing number of studies related to cerebral ischemic injury and repair have shifted their focus from neurons to the NVU [54]. A transcriptome analysis revealed increased expression of *Spp1*, encoding osteopontin, in all cells of the NVU during the acute phase of ischemic stroke. Subcutaneous administration of anti-osteopontin antibodies after ischemic stroke in mice promotes BBB preservation, reduces cerebral edema, and decreases the risk of hemorrhagic transformation, leading to an improved prognosis [96]. BMECs deliver diverse functional messages to neural tissue via exosomes and extracellular vesicles (EVs). Caveolae in arteriolar ECs transmit neuronal signals to cerebral smooth muscle cells, where they participate in vasodilation regulation [97]. A recent study showed that following OGD, BMECs release microvesicles that transfer Ascl1 (a proneural transcription factor) into astrocytes, converting them into neural progenitors. This endogenous trans-differentiation provides a novel therapeutic perspective for ischemic stroke [98].

Under ischemic conditions, BMECs exert direct neuroprotective effects by delivering oxygen and glucose to neurons and secreting neurotrophic factors [99]. Notably, almost all the BMECs are surrounded by astrocyte endfeet, which allow BMECs to interact with the neurons [100]. The activation of VEGF by astrocytes represents a crucial pathway for protecting neural and vascular homeostasis under ischemic and hypoxic conditions [101]. VEGF not only acts as an endothelial growth factor to increase the antioxidant capacity of BMECs and attenuate cellular damage [101] but also promotes synaptogenesis and improves locomotor function. For example, the depletion of VEGF in BMECs abolishes their ability to increase excitatory postsynaptic currents in the motor cortex upon implantation [102]. Furthermore, VEGF also rapidly suppresses glutamate- and hypoxia-induced calcium influx into astrocytes, which contributes to the development of ischemic brain injury [103]. In addition to VEGF, astrocyte-derived factors that regulate BBB impairment and repair after brain injury have been reviewed [104]. Therefore, within the NVU, BMECs play a role that extends far beyond passively maintaining the BBB; they actively contribute to CNS signaling through the secretion of neurotrophic factors or vesicles. In this process, astrocytes act as critical signaling intermediaries, orchestrating communication among various NVU components, whereas pericytes primarily function to rapidly respond to regulatory signals from BMECs and astrocytes, modulating CBF through contraction and relaxation. Although the current understanding of how to harness this intercellular synergy within the NVU to promote neurovascular protection under ischemic/hypoxic conditions remains limited, it holds promising potential as a key therapeutic target for improving cerebral perfusion and enhancing neuroprotection.

### 3.4 Dysregulation of the BMEC transporter under ischemic/hypoxic conditions

In ischemic brain injury, reduced CBF induces BBB transporter dysregulation through the following mechanisms: glucose transporter dysfunction, which destabilizes energy metabolism; ion/water imbalance coupled with glutamate transporter impairment, which exacerbates cerebral edema; and efflux transporter dysfunction, which increases drug toxicity [14]. During cerebral ischemia, oxygen and nutrient deprivation increase energy demands in the brain, and the upregulation of GLUTs increases the cerebral glucose supply [105]. This process is governed by the hypoxia regulator Vav1. Hypoxia inhibits Vav1 degradation, promoting the accumulation and stabilization of Vav1-regulated HIF-1α, which further induces GLUT1 transcription, thereby regulating glucose metabolism in the brain [106]. During early ischemic stroke, cerebral edema correlates with increased sodium transporter activity. Ischemia enhances Na-K-Cl cotransport and Na/H exchange in BMECs, triggering elevated Na^+^, Cl_-_, and water secretion into brain tissue and subsequent edema formation [107]. The blockade of KCa3.1 (calcium-activated potassium channels) mitigates both Na^+^ uptake and cytotoxic edema in ischemic brains [35]. In addition, zinc homeostasis critically sustains BBB integrity. Zinc transporter 4 (ZnT4) facilitates zinc exocytosis from the cytoplasm to maintain appropriate intracellular zinc concentrations. Under OGD conditions, miR-30a negatively regulates ZnT4 protein levels, increasing free zinc levels in BMECs and mediating BBB damage. Therefore, miR-30a inhibition attenuates ischemia-induced barrier disruption via the ZnT4 pathway; thus, this miRNA is a target for improving the prognosis of cerebral infarction [108].

P-gp, an ATP-binding cassette (ABC) transporter family member, functions as a ubiquitous efflux pump that mediates the clearance of diverse exogenous toxins and drugs [109]. The modulation of the P-gp protein is necessary to allow drug transport to the CNS and prevent toxic substances from entering the brain. HMGB1 induces inflammatory responses via the TLR4/NF-κB pathway and participates in the upregulation of P-gp in BMECs during ischemic stroke [110]. After cerebral ischemic injury, elevated P-gp expression inhibits AKT/mTOR-induced endothelial autophagy, resulting in increased BBB permeability. Silencing P-gp expression restores TJ protein expression and attenuates barrier dysfunction, thereby reducing brain damage and improving neurological function [111]. In summary, ischemia and hypoxia induce the concerted dysregulation of multiple critical transport proteins, encompassing impaired glucose metabolism, the disruption of ion and water homeostasis, and the accumulation of neurotoxic substances, which collectively exacerbate brain injury. Current therapeutic strategies targeting the transport barrier focus primarily on restoring the function of transport proteins and reestablishing homeostasis. Future research assessing multitarget interventions for these interconnected transport processes holds promise for the development of more systematic and effective neuroprotective strategies for ischemic brain injury.

### 3.5 The involvement of BMECs in the ischemia/hypoxia-induced inflammatory response

Inflammatory processes significantly contribute to BBB disruption. Postischemic activation of microglia and astrocytes triggers the substantial release of inflammatory mediators and proteases, subsequently compromising barrier integrity [112]. VEGF-A/VEGFR-2 signaling and the neuronal NLPR3 inflammasome activate inflammatory signaling cascades in BMECs and exacerbate cerebrovascular leakage [113, 114], promoting the accumulation of toxic substances in the brain and inducing neuroinflammation and neurological dysfunction [15]. Under normal conditions, vesicle-mediated transcytosis in BMECs is inhibited to maintain BBB integrity. However, when an ischemic stroke occurs, vesicle-mediated transcytosis is activated, which is another cause of disrupted BBB integrity [97]. Moreover, BBB disruption after stroke also upregulates the expression of adhesion molecules, which promote leukocyte migration across the BBB through paracellular and/or transcellular diapedesis, leading to CNS inflammatory responses [115]. As mentioned, PECAM-1 is highly expressed on leukocytes and BMECs and is a microvesicular protein that is essential for transendothelial migration (TEM). PECAM-1-positive neutrophils migrate across the endothelium to infiltrate the ischemic cerebral hemisphere and promote neuroinflammation after ischemic stroke. Neutrophil TEM is suppressed when *Pecam-1* is knocked down; thus, *Pecam-1* is one of the targets for controlling neuroinflammation after stroke [116]. miRNAs critically modulate inflammation-driven changes in gene expression in BMECs. miR-98 expression is reduced in response to ischemia/hypoxia and inflammation. miR-98 overexpression limits proinflammatory leukocyte migration into the brain and prevents microglial activation, attenuating inflammatory responses [117].

Although neuroinflammation leads to brain damage in acute-phase ischemic stroke, immune cells facilitate late recovery by promoting angiogenesis, BBB repair, and neurogenesis [118]. In the later stages of injury, microglia acquire a protective phenotype and participate in repair mechanisms, including postinjury angiogenesis [115]. EVs from OGD-induced microglia are enriched in TGF-β1, driving the M2 polarization of resident microglia in ischemic brains, which inhibits neuronal cell injury and stimulates angiogenesis and tube formation [119]. Brain ECs exhibit elevated levels of ABCB1 under OGD conditions. Neural progenitor cell (NPC)-derived EVs reduce ABCB1 expression and inhibit NF-κB signaling and subsequent MMP-9 activation to reduce inflammatory cell recruitment, thereby promoting BBB repair after stroke [120]. Therefore, the immune response in ischemic stroke plays both detrimental and reparative roles in a time-dependent and cell-specific manner. During the acute phase, excessive immune activation contributes to BBB disruption and neuronal damage. In contrast, in the recovery phase, the immune response supports tissue repair, angiogenesis, and neurogenesis through the phenotypic switching of microglia and the secretion of protective factors. Recent single-cell studies have revealed that ischemic stroke induces spatially and transcriptionally distinct microglial subpopulations in which ischemic core-associated microglia drive pathological inflammation, whereas ischemic penumbra-associated microglia perform neuroprotective and reparative functions [121]. This spatiotemporal and functional complexity underscores the potential for precisely targeted immunomodulatory therapies—not only to suppress early destructive inflammation but also to harness these repair mechanisms to improve long-term outcomes after ischemic stroke.

### 3.6 Coculture of BMECs with non-NVU cells promotes the repair of brain injury after ischemia

Comparative studies of HBMECs versus outgrowth endothelial cells (OECs) have shown that OECs exhibit lower NADPH oxidase activity and a greater total antioxidant capacity to effectively restore BBB integrity after ischemic injury during OGD and OGD/R [122]. In one study, cotreatment of a BBB model with OEC-derived conditioned media counteracted the TNF-α-induced barrier compromise [11]. EVs secreted by mesenchymal stem cells also affect BMECs. EVs can induce BMEC angiogenesis by promoting the formation of tubules. EVs from hypoxic cultures have a more dramatic effect because they further upregulate the expression of angiogenic molecules and TJ proteins [123]. Furthermore, coculture of endothelial progenitor cells and NPCs synergistically inhibits hypoxia/reoxygenation (H/R)-induced ROS overproduction, which is mediated primarily by VEGF and BDNF through paracrine signaling. The enhanced angiogenic and barrier functions observed under coculture conditions protect cerebral ECs from H/R injury [124].

## 4. The protective effect of ischemia/hypoxia conditioning is mediated by modulating the diversity of BMECs

The core cause of BMEC injury-induced cerebral microvascular dysfunction in ischemic stroke models is ischemia/hypoxia. Enhancing cerebral ischemia/hypoxia tolerance to regulate BMECs represents a critical approach for restoring microvascular heterogeneity and ultimately improving the disease prognosis [125]. Ischemia/hypoxia conditioning, a sublethal ischemic/hypoxic event in which a short period of mild ischemic/hypoxic stimulation is administered to an organism, can significantly increase the tolerance of that organism to subsequent more severe ischemic/hypoxic events [126]. Ischemia/hypoxia conditioning is an extensively investigated neuroprotective strategy for treating CNS diseases [127]. This review focuses on its protective effect on BMECs (Table 1).

Remote ischemic conditioning (RIC) is an intrinsically protective phenomenon that protects critical organs such as the brain, heart, and kidneys from sustained I/R injury through 3-4 cycles of nonlethal local ischemia with reperfusion to distal tissues [128]. Since approximately 1990, ischemic preconditioning has been reported as a neuroprotective intervention for cerebral infarction [99]. In addition to its neuroprotective effects [126], RIC also has protective effects on cerebral microvessels and BMECs during ischemic stroke. *In vitro* studies revealed that EVs released after RIC increase the viability of HBMECs subjected to OGD [129]. *In vivo*, RIC promotes arteriogenesis, improves CBF [130], increases the microvessel density [131], and promotes collateral vessel formation [132] in an ischemic stroke model. RIC also maintains BBB integrity after stroke. A recent study revealed that RIC protects barrier integrity, increases the expression of TJ proteins and GLUT1, and decreases the expression of VCAM1 in APP/PS1 rats [133]. Recombinant tissue plasminogen activator (rt-PA) therapy for acute cerebral infarction is associated with BBB disruption and increased hemorrhagic conversion. However, RIC attenuates the rt-PA-mediated exacerbation of BBB disruption by reducing the activity of the PDGF-CC/PDGFRα pathway [134]. Other studies have combined physical activity with RIC during stroke rehabilitation and reported that, compared with either RIC or physical activity alone, the combination of the two improves functional outcomes, synaptogenesis, and angiogenesis after stroke and has better efficacy in stroke rehabilitation [135, 136]. Beyond its established functions in acute cerebrovascular disease, RIC has been shown to promote angiogenesis [137], improve CBF, and promote collateral circulation [138] in a chronic cerebral hypoperfusion model.

**Table 1:** Protective effects of ischemia/hypoxia conditioning on BMECs.

References	RIC/HC	Pattern	In vivo/ in vitro	Model	Protective effects on endothelial cells or brain microvessels	Signal pathway
**Gu et al.^[129]^**	RIC	5 min ischemia + 5 min reperfusion, 4 cycles, 3 times a week, 6 weeks	In vitro	Oxygen and glucose deprivation (endothelial cells)	Improving viability of endothelial cells	N
**Ren et al.^[130]^**	RIC	10 min ischemia + 10 min reperfusion, 3 cycles, once a day, 7/14 days	In vivo	Rat 90 minutes of MCAO model	Promoting angiogenesis; improving CBF; promoting collateral formation	Notch signal pathway
**Liang et al.^[131]^**	RIC	10 min ischemia + 10 min reperfusion, 3 cycles, once a day, 21 days	In vivo	Rat 90 minutes of MCAO model	Increasing microvessel density	Increasing tissue kallirein level in blood
**Saito et al.^[132]^**	RIC	5 min ischemia + 5 min reperfusion, 4 cycles, once a day, 1/2 days	In vivo	Mouse permanent MCAO model	Promoting collateral formation	AKT/eNOS signal pathway
**Ma et al.^[133]^**	RIC	10 min ischemia + 10 min reperfusion, 4 cycles, 5 times a week, 30 days	In vivo	*APP/ PS1*-Tg rats	Increasing CBF; protecting BBB integrity	N
**He et al.^[134]^**	RIC	5 min ischemia + 5 min reperfusion, 4 cycles, twice a day RIPreC: 7 days; RIPostC: 3 days	In vivo	Rat thromboembolic stroke model	Attenuating rtPA-aggravated BBB disruption	Reducing the PDGF-CC/PDGFRα pathway
**Wang et al.^[135]^**	RIC	10 min ischemia + 10 min reperfusion, 3 cycles, once a day, 28 days	In vivo	Rat focal cerebral ischemia model	Promoting angiogenesis	Inhibiting the NLRP3 inflammasome
**Geng et al.^[136]^**	RIC	10 min ischemia + 10 min reperfusion, 3 cycles, once a day, 28 days	In vivo	Rat focal cerebral ischemia model	Promoting angiogenesis; increasing angiogenic factors, including VEGF, Ang-1, and Ang-2	HIF-1α signal pathway
**Ren et al.^[137]^**	RIC	10 min ischemia + 10 min reperfusion, 3 cycles, once a day, 28 days	In vivo	Rat chronic cerebral hypoperfusion model	Promoting angiogenesis	eNOS/NO signal pathway
**Khan et al.^[138]^**	RIC	5 min ischemia + 5 min reperfusion, 4 cycles, once a day, 1 month or 4 months	In vivo	Mouse bilateral carotid artery stenosis model	Promoting angiogenesis; improving CBF; promoting new collateral formation	N
**Ou et al.^[140]^**	HC	1% O_2_, 94% N_2_, and 5% CO_2_ hypoxia (1, 2 or 4 hours) + 95% air and 5% CO_2_ reoxygenation (24 hours)	In vitro	1% O_2_, 94% N_2_, and 5% CO_2_ hypoxia (48 hours)	Stabilizing endothelial cells	Inhibiting the activation of HIF-1α and its regulation of downstream VEGF and ANGPTL-4
**Guan et al.^[141]^**	HC	5 min reoxygenation (21% O_2_) + 5 min hypoxia (13% O_2_), 10 cycles, once a day, 14 days	In vivo	Mouse distal MCAO model	Promoting angiogenesis	N
**Jiang et al.^[142]^**	HC	3% O2, 5% CO2, 92% N2 (12 hours, conditioned medium derived from bone marrow mesenchymal stromal cells)	In vivo	Rat MCAO model	Promoting angiogenesis	PI3K/AKT signal pathway
**Stowe et al.^[143]^**	HC	9 times hypoxic exposures in 15 days (8% or 11% O_2_), 2 or 4 hours	In vivo	Mouse 60-min transient MCAO model	Reducing vascular inflammation, maintaining BBB integrity	N

*RIC, remote ischemic conditioning; MCAO: middle cerebral artery occlusion; RIPostC, remote ischemic post-conditioning; RIPreC, remote ischemic preconditioning; HC, hypoxic conditioning; BBB, blood brain barrier; CBF, cerebral blood flow; N, not mentioned.

Intermittent hypoxia is a safe, noninvasive, easy-to-administer systemic intervention featuring short-term normobaric hypoxia/normoxia/hyperoxia alternations that promote adaptive responses and ameliorate the deleterious effects of persistent hypoxia [139]. HIF-1α serves as the master regulator of hypoxic/ischemic responses and mediates the hypoxic adaptation. HIF-1α protein stabilization under hypoxic conditions is the cellular adaptation to oxygen deprivation. Cell type-specific adaptive capacities to hypoxic/ischemic stress vary significantly. BMECs exhibit high sensitivity to oxygen deprivation, whereas astrocytes and pericytes tolerate longer periods of hypoxia. The rapid and significant increase in endothelial HIF-1α expression under hypoxic conditions is consistent with the finding that BMECs are highly sensitive to oxygen deprivation [40]. Hypoxic preconditioning can exert a potent protective effect on retinal microvascular ECs by suppressing HIF-1α-mediated VEGF and ANGPTL4 signaling [140]. In a distal MCAO model, intermittent hypoxia treatment increases the capillary length and volume, increases the number of collateral vessels, and effectively induces angiogenesis in the mouse brain [141]. Hypoxia-preconditioned bone marrow mesenchymal stromal cell-conditioned medium markedly reduces the cerebral infarct volume and enhances functional recovery in MCAO rats, which correlates with the suppression of neuronal apoptosis and stimulation of angiogenesis [142]. Hypoxic preconditioning also modulates the inflammatory response after cerebral ischemic injury. Hypoxic preconditioning reduces leukocyte extravasation and decreases BBB permeability to endogenous IgG, thereby attenuating poststroke inflammatory responses [143].

### Limitations and prospects

Current research on the physiological functions of the cerebral microvasculature has focused primarily on coordinating angiogenesis, maintaining BBB integrity, and regulating substance transport. These foundational studies have laid the crucial groundwork for understanding microvascular homeostasis, with landmark findings of the role of BMECs in junctional dynamics, transporter specificity, and angiogenic signaling. However, the cerebral microvasculature operates as a dynamic functional network rather than a collection of isolated modules. The ability of the brain microvasculature to interact with neurons to match metabolic demands via neurovascular coupling, paracrine signaling, and synaptic modulation represents an equally vital dimension that remains underexplored. Furthermore, the role of BMECs as a bridge between central and peripheral immunity—governing immune cell trafficking while integrating systemic signals—highlights another critical node in this network and underscores the need for future studies to map these cross-system interactions.

Despite significant progress in elucidating the mechanisms of cerebrovascular damage, the intrinsic adaptive potential of BMECs has yet to be fully harnessed. Angiogenesis, a well-recognized self-rescue postinjury mechanism, has been validated by numerous studies to restore perfusion. However, its therapeutic efficacy is limited by a failure to synchronize with BBB repair, resulting in a functional mismatch: an increased vessel density without reduced permeability fails to restore microvascular homeostasis. This paradox emphasizes that future interventions must move beyond single-target strategies. Rather than solely promoting angiogenesis or stabilizing the BBB in isolation, therapies should aim to rebalance the entire microvascular network: coordinating angiogenic sprouting with junctional integrity, nutrient transport, and immune quiescence. Such an approach could unlock the full potential of BMEC-mediated repair, particularly in ischemic/hypoxic conditions where multifaceted dysfunction predominates.

Ischemia/hypoxia conditioning, a promising strategy validated by robust evidence of its protective effects on BMEC survival, angiogenesis, and BBB preservation, represents a prime candidate for this network-based optimization (Fig. 3). Existing studies have provided invaluable insights into its ability to mitigate neurological impairments, as landmark research on RIC and intermittent hypoxia has shown its capacity to increase microvascular resilience. However, the current research primarily evaluates isolated endpoints, leaving critical gaps in our understanding of how conditioning modulates the entire functional network. Future studies should prioritize defining how conditioning protocols with different spatial scales (local, remote, or systemic) and temporal patterns systemically regulate these network nodes. This shift toward network-targeted conditioning could improve therapeutic outcomes and ensure that the protective effects on BMECs translate into comprehensive microvascular recovery, which ultimately improves the clinical prognosis of patients with ischemic cerebrovascular disease. However, the clinical translation of ischemia/hypoxia conditioning still faces several limitations. First, the primary patient population with ischemic cerebrovascular disease is elderly; thus, age is a significant risk factor that cannot be overlooked. Current studies use adult rather than aged animals for modeling; incorporating aged animals would better simulate the clinical scenario. Second, elderly patients are often present with more comorbidities. The lack of comprehensive models incorporating these risk factors also contributes to the difficulty in clinical translation. Third, conditioning protocols vary considerably across studies. Although preconditioning may be beneficial for disease prevention, its clinical application is hampered by poor patient compliance. While postconditioning could improve patient retention, its parameters, such as the timing of initiation, treatment duration, and specific regimen, exhibit substantial variability among studies. Currently, no unified evaluation standard is available to normalize treatment outcomes across different investigations.

**Figure 3.**
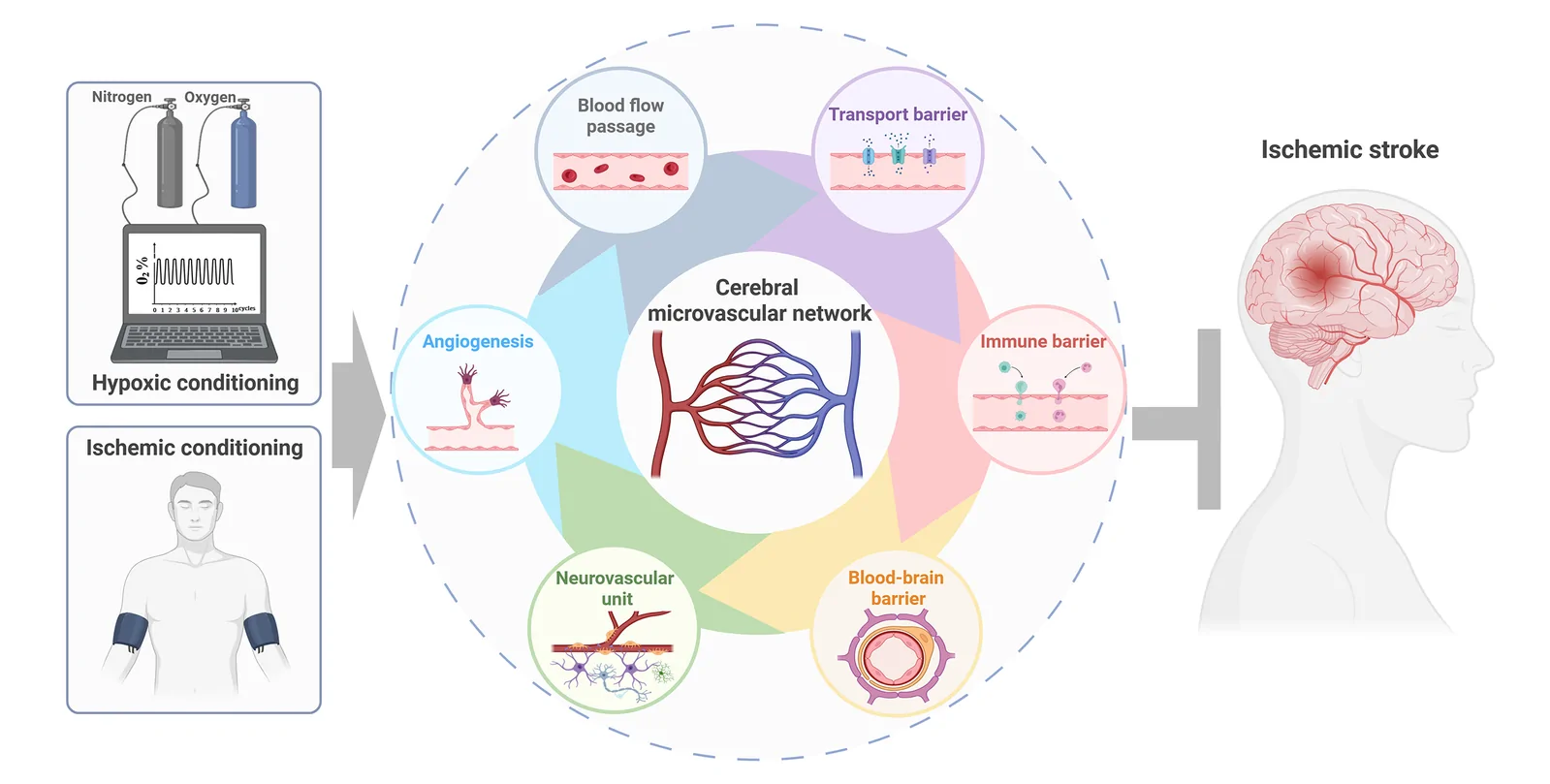
**Ischemia/hypoxia conditioning as a candidate for targeting the entire cerebral microvascular network**. Ischemia/hypoxia conditioning potentially promotes angiogenesis, improves blood flow, modulates transport across barriers, regulates immune responses, improves blood-brain barrier function, and enhances neurovascular unit function. Thus, this conditioning contributes to the overall improvement of the cerebral microvascular network and represents a highly promising therapeutic strategy for ischemic cerebrovascular diseases.

### Conclusions

ECs play important roles in the vasculature of various organs throughout the body. As highly specialized ECs in the brain, BMECs play an indispensable and unique role in CNS. Ischemia/hypoxia-induced brain injury is associated with disruptions of BBB and NVU homeostasis. BMECs also play important roles in maintaining homeostasis and in damage repair. In this review, the role of ischemia/hypoxia conditioning in the targeted therapy of BMECs is described. This knowledge contributes to the development of early interventions for ischemic cerebrovascular disease. We already know that early protection of BMECs is beneficial for maintaining the integrity of the BBB and NVU; thus, further exploration of protective measures for BMECs is necessary. Ischemia/hypoxia conditioning is a promising therapeutic approach that should be explored in future studies.

## References

[1] Webb AJS, Werring DJ (2022). New Insights Into Cerebrovascular Pathophysiology and Hypertension. Stroke, 53:1054-1064. 10.1161/STROKEAHA.121.035850 PMC7615037 35255709

[2] Xu K, Du W, Zhuang X, Liang D, Mo Y, Wang J (2023). Glycogen synthase kinase-3β mediates toll-like receptors 4/nuclear factor kappa-B-activated cerebral ischemia-reperfusion injury through regulation of fat mass and obesity-associated protein. Brain Circ, 9:162-171. 10.4103/bc.bc_3_23 PMC10679630 38020949

[3] Tudor T, Spinazzi EF, Alexander JE, Mandigo GK, Lavine SD, Grinband J, et al. (2024). Progressive microvascular failure in acute ischemic stroke: A systematic review, meta-analysis, and time-course analysis. J Cereb Blood Flow Metab, 44:192-208. 10.1177/0271678X231216766 PMC10993872 38016953

[4] Guan Y, Liu J, Gu Y, Ji X (2023). Effects of Hypoxia on Cerebral Microvascular Angiogenesis: Benefits or Damages^+^ Aging Dis, 14:370-385. 10.14336/AD.2022.0902 PMC10017152 37008044

[5] Shigemoto-Mogami Y, Nakayama-Kitamura K, Sato K (2024). The arrangements of the microvasculature and surrounding glial cells are linked to blood-brain barrier formation in the cerebral cortex. Front Neuroanat, 18:1438190. 10.3389/fnana.2024.1438190 PMC11335649 39170850

[6] Hu Y, Zheng Y, Wang T, Jiao L, Luo Y (2022). VEGF, a Key Factor for Blood Brain Barrier Injury After Cerebral Ischemic Stroke. Aging Dis, 13:647-654. 10.14336/AD.2021.1121 PMC9116914 35656098

[7] Jamieson JJ, Lin Y, Malloy N, Soto D, Searson PC, Gerecht S (2022). Hypoxia-induced blood-brain barrier dysfunction is prevented by pericyte-conditioned media via attenuated actomyosin contractility and claudin-5 stabilization. Faseb j, 36:e22331. 10.1096/fj.202200010RR PMC9060394 35476363

[8] Chen Z, Li G (2021). Immune response and blood-brain barrier dysfunction during viral neuroinvasion. Innate Immun, 27:109-117. 10.1177/1753425920954281 PMC7882805 32903111

[9] Gorina R, Lyck R, Vestweber D, Engelhardt B (2014). β2 integrin-mediated crawling on endothelial ICAM-1 and ICAM-2 is a prerequisite for transcellular neutrophil diapedesis across the inflamed blood-brain barrier. J Immunol, 192:324-337. 10.4049/jimmunol.1300858 24259506

[10] Page S, Raut S, Al-Ahmad A (2019). Oxygen-Glucose Deprivation/Reoxygenation-Induced Barrier Disruption at the Human Blood-Brain Barrier is Partially Mediated Through the HIF-1 Pathway. Neuromolecular Med, 21:414-431. 10.1007/s12017-019-08531-z 30911877

[11] Kadir RRA, Alwjwaj M, Rakkar K, Othman OA, Sprigg N, Bath PM, et al. (2023). Outgrowth Endothelial Cell Conditioned Medium Negates TNF-α-Evoked Cerebral Barrier Damage: A Reverse Translational Research to Explore Mechanisms. Stem Cell Rev Rep, 19:503-515. 10.1007/s12015-022-10439-4 PMC9902316 36056287

[12] Warpsinski G, Smith MJ, Srivastava S, Keeley TP, Siow RCM, Fraser PA, et al. (2020). Nrf2-regulated redox signaling in brain endothelial cells adapted to physiological oxygen levels: Consequences for sulforaphane mediated protection against hypoxia-reoxygenation. Redox Biol, 37:101708. 10.1016/j.redox.2020.101708 PMC7502377 32949969

[13] Miller J, Moloney JA, Elagamy N, Tuttle J, Tirgari S, Calo S, et al. (2024). Cerebral blood flow change with fluid resuscitation in acute ischemic stroke. Brain Circ, 10:303-307. 10.4103/bc.bc_30_24 PMC11850932 40012591

[14] Sifat AE, Vaidya B, Villalba H, Albekairi TH, Abbruscato TJ (2019). Neurovascular unit transport responses to ischemia and common coexisting conditions: smoking and diabetes. Am J Physiol Cell Physiol, 316:C2-C15. 10.1152/ajpcell.00187.2018 PMC6383146 30207783

[15] Freitas-Andrade M, Raman-Nair J, Lacoste B (2020). Structural and Functional Remodeling of the Brain Vasculature Following Stroke. Front Physiol, 11:948. 10.3389/fphys.2020.00948 PMC7433746 32848875

[16] Eelen G, de Zeeuw P, Treps L, Harjes U, Wong BW, Carmeliet P (2018). Endothelial Cell Metabolism. Physiol Rev, 98:3-58. 10.1152/physrev.00001.2017 PMC5866357 29167330

[17] Dias MC, Quesada AO, Soldati S, Bösch F, Gruber I, Hildbrand T, et al. (2021). Brain endothelial tricellular junctions as novel sites for T cell diapedesis across the blood-brain barrier. J Cell Sci, 134:jcs253880. 10.1242/jcs.253880 PMC8121105 33912914

[18] Longden TA, Dabertrand F, Koide M, Gonzales AL, Tykocki NR, Brayden JE, et al. (2017). Capillary K(+)-sensing initiates retrograde hyperpolarization to increase local cerebral blood flow. Nat Neurosci, 20:717-726. 10.1038/nn.4533 PMC5404963 28319610

[19] Choublier N, Taghi M, Menet MC, Le Gall M, Bruce J, Chafey P, et al. (2022). Exposure of human cerebral microvascular endothelial cells hCMEC/D3 to laminar shear stress induces vascular protective responses. Fluids Barriers CNS, 19:41. 10.1186/s12987-022-00344-w PMC9164338 35658915

[20] Garcia-Polite F, Martorell J, Del Rey-Puech P, Melgar-Lesmes P, O'Brien CC, Roquer J, et al. (2017). Pulsatility and high shear stress deteriorate barrier phenotype in brain microvascular endothelium. J Cereb Blood Flow Metab, 37:2614-2625. 10.1177/0271678X16672482 PMC5531355 27702879

[21] van der Wijk AE, Georgakopoulou T, Majolée J, van Bezu JSM, van der Stoel MM, van Het Hof BJ, et al. (2020). Microembolus clearance through angiophagy is an auxiliary mechanism preserving tissue perfusion in the rat brain. Acta Neuropathol Commun, 8:195. 10.1186/s40478-020-01071-9 PMC7671188 33203478

[22] Lam CK, Yoo T, Hiner B, Liu Z, Grutzendler J (2010). Embolus extravasation is an alternative mechanism for cerebral microvascular recanalization. Nature, 465:478-482. 10.1038/nature09001 PMC2879083 20505729

[23] Lee HW, Xu Y, He L, Choi W, Gonzalez D, Jin SW, et al. (2021). Role of Venous Endothelial Cells in Developmental and Pathologic Angiogenesis. Circulation, 144:1308-1322. 10.1161/CIRCULATIONAHA.121.054071 PMC9153651 34474596

[24] Ben-Zvi A, Liebner S (2022). Developmental regulation of barrier- and non-barrier blood vessels in the CNS. J Intern Med, 292:31-46. 10.1111/joim.13263 33665890

[25] Crouch EE, Bhaduri A, Andrews MG, Cebrian-Silla A, Diafos LN, Birrueta JO, et al. (2022). Ensembles of endothelial and mural cells promote angiogenesis in prenatal human brain. Cell, 185:3753-3769.e18. 10.1016/j.cell.2022.09.004 PMC9550196 36179668

[26] Naito H, Iba T, Takakura N (2020). Mechanisms of new blood-vessel formation and proliferative heterogeneity of endothelial cells. Int Immunol, 32:295-305. 10.1093/intimm/dxaa008 31996897

[27] Adams RH, Alitalo K (2007). Molecular regulation of angiogenesis and lymphangiogenesis. Nat Rev Mol Cell Biol, 8:464-478. 10.1038/nrm2183 17522591

[28] Banki E, Sosnowska D, Tucsek Z, Gautam T, Toth P, Tarantini S, et al. (2015). Age-related decline of autocrine pituitary adenylate cyclase-activating polypeptide impairs angiogenic capacity of rat cerebromicrovascular endothelial cells. J Gerontol A Biol Sci Med Sci, 70:665-674. 10.1093/gerona/glu116 PMC4447801 25136000

[29] Zhuang SF, Liu DX, Wang HJ, Zhang SH, Wei JY, Fang WG, et al. (2017). Atg7 Regulates Brain Angiogenesis via NF-κB-Dependent IL-6 Production. Int J Mol Sci, 18:968. 10.3390/ijms18050968 PMC5454881 28467355

[30] He L, Wei JY, Liu DX, Zhao WD, Chen YH (2019). Atg7 Silencing Inhibits Laminin-5 Expression to Suppress Tube Formation by Brain Endothelial Cells. Anat Rec (Hoboken), 302:2255-2260. 10.1002/ar.24223 31265765

[31] Anasooya Shaji C, Robinson BD, Yeager A, Beeram MR, Davis ML, Isbell CL, et al. (2019). The Tri-phasic Role of Hydrogen Peroxide in Blood-Brain Barrier Endothelial cells. Sci Rep, 9:133. 10.1038/s41598-018-36769-3 PMC6333800 30644421

[32] Wälchli T, Pernet V, Weinmann O, Shiu JY, Guzik-Kornacka A, Decrey G, et al. (2013). Nogo-A is a negative regulator of CNS angiogenesis. Proc Natl Acad Sci U S A, 110:E1943-1952. 10.1073/pnas.1216203110 PMC3666688 23625008

[33] Andjelkovic AV, Situ M, Citalan-Madrid AF, Stamatovic SM, Xiang J, Keep RF (2023). Blood-Brain Barrier Dysfunction in Normal Aging and Neurodegeneration: Mechanisms, Impact, and Treatments. Stroke, 54:661-672. 10.1161/STROKEAHA.122.040578 PMC9993074 36848419

[34] Archie SR, Al Shoyaib A, Cucullo L (2021). Blood-Brain Barrier Dysfunction in CNS Disorders and Putative Therapeutic Targets: An Overview. Pharmaceutics, 13:1779. 10.3390/pharmaceutics13111779 PMC8622070 34834200

[35] Chen YJ, Wallace BK, Yuen N, Jenkins DP, Wulff H, O'Donnell ME (2015). Blood-brain barrier KCa3.1 channels: evidence for a role in brain Na uptake and edema in ischemic stroke. Stroke, 46:237-244. 10.1161/STROKEAHA.114.007445 PMC4276511 25477223

[36] Clyne AM (2021). Endothelial response to glucose: dysfunction, metabolism, and transport. Biochem Soc Trans, 49:313-325. 10.1042/BST20200611 PMC7920920 33522573

[37] Chiba H, Ichikawa-Tomikawa N, Imura T, Sugimoto K (2021). The region-selective regulation of endothelial claudin-5 expression and signaling in brain health and disorders. J Cell Physiol, 236:7134-7143. 10.1002/jcp.30357 33694168

[38] Engelhardt S, Patkar S, Ogunshola OO (2014). Cell-specific blood-brain barrier regulation in health and disease: a focus on hypoxia. Br J Pharmacol, 171:1210-1230. 10.1111/bph.12489 PMC3952799 24641185

[39] Wang X, Xu B, Xiang M, Yang X, Liu Y, Liu X, et al. (2020). Advances on fluid shear stress regulating blood-brain barrier. Microvasc Res, 128:103930. 10.1016/j.mvr.2019.103930 31639383

[40] Engelhardt S, Huang SF, Patkar S, Gassmann M, Ogunshola OO (2015). Differential responses of blood-brain barrier associated cells to hypoxia and ischemia: a comparative study. Fluids Barriers CNS, 12:4. 10.1186/2045-8118-12-4 PMC4429667 25879623

[41] Inoue Y, Shue F, Bu G, Kanekiyo T (2023). Pathophysiology and probable etiology of cerebral small vessel disease in vascular dementia and Alzheimer's disease. Mol Neurodegener, 18:46. 10.1186/s13024-023-00640-5 PMC10334598 37434208

[42] Bader A, Bintig W, Begandt D, Klett A, Siller IG, Gregor C, et al. (2017). Adenosine receptors regulate gap junction coupling of the human cerebral microvascular endothelial cells hCMEC/D3 by Ca(2+) influx through cyclic nucleotide-gated channels. J Physiol, 595:2497-2517. 10.1113/JP273150 PMC5390872 28075020

[43] Yan L, Moriarty RA, Stroka KM (2021). Recent progress and new challenges in modeling of human pluripotent stem cell-derived blood-brain barrier. Theranostics, 11:10148-10170. 10.7150/thno.63195 PMC8581424 34815809

[44] Megra BW, Eugenin EA, Berman JW (2018). Inflammatory mediators reduce surface PrP(c) on human BMVEC resulting in decreased barrier integrity. Lab Invest, 98:1347-1359. 10.1038/s41374-018-0090-z PMC6163073 29959417

[45] Ito S, Yanai M, Yamaguchi S, Couraud PO, Ohtsuki S (2017). Regulation of Tight-Junction Integrity by Insulin in an In Vitro Model of Human Blood-Brain Barrier. J Pharm Sci, 106:2599-2605. 10.1016/j.xphs.2017.04.036 28456720

[46] Rom S, Zuluaga-Ramirez V, Dykstra H, Reichenbach NL, Ramirez SH, Persidsky Y (2015). Poly(ADP-ribose) polymerase-1 inhibition in brain endothelium protects the blood-brain barrier under physiologic and neuroinflammatory conditions. J Cereb Blood Flow Metab, 35:28-36. 10.1038/jcbfm.2014.167 PMC4294393 25248836

[47] Zlotnik D, Rabinski T, Halfon A, Anzi S, Plaschkes I, Benyamini H, et al. (2022). P450 oxidoreductase regulates barrier maturation by mediating retinoic acid metabolism in a model of the human BBB. Stem Cell Reports, 17:2050-2063. 10.1016/j.stemcr.2022.07.010 PMC9481905 35961311

[48] Tsai PS, Kaufhold JP, Blinder P, Friedman B, Drew PJ, Karten HJ, et al. (2009). Correlations of neuronal and microvascular densities in murine cortex revealed by direct counting and colocalization of nuclei and vessels. J Neurosci, 29:14553-14570. 10.1523/JNEUROSCI.3287-09.2009 PMC4972024 19923289

[49] Segarra M, Aburto MR, Hefendehl J, Acker-Palmer A (2019). Neurovascular Interactions in the Nervous System. Annu Rev Cell Dev Biol, 35:615-635. 10.1146/annurev-cellbio-100818-125142 31590587

[50] Zhao Y, Yang J, Li C, Zhou G, Wan H, Ding Z, et al. (2020). Role of the neurovascular unit in the process of cerebral ischemic injury. Pharmacol Res, 160:105103. 10.1016/j.phrs.2020.105103 32739425

[51] Thakore P, Alvarado MG, Ali S, Mughal A, Pires PW, Yamasaki E, et al. (2021). Brain endothelial cell TRPA1 channels initiate neurovascular coupling. Elife, 10:e63040. 10.7554/eLife.63040 PMC7935492 33635784

[52] Negri S, Faris P, Soda T, Moccia F (2021). Endothelial signaling at the core of neurovascular coupling: The emerging role of endothelial inward-rectifier K(+) (K(ir)2.1) channels and N-methyl-d-aspartate receptors in the regulation of cerebral blood flow. Int J Biochem Cell Biol, 135:105983. 10.1016/j.biocel.2021.105983 33894355

[53] Alarcon-Martinez L, Villafranca-Baughman D, Quintero H, Kacerovsky JB, Dotigny F, Murai KK, et al. (2020). Interpericyte tunnelling nanotubes regulate neurovascular coupling. Nature, 585:91-95. 10.1038/s41586-020-2589-x 32788726

[54] Cai W, Zhang K, Li P, Zhu L, Xu J, Yang B, et al. (2017). Dysfunction of the neurovascular unit in ischemic stroke and neurodegenerative diseases: An aging effect. Ageing Res Rev, 34:77-87. 10.1016/j.arr.2016.09.006 PMC5384332 27697546

[55] Fu J, Li L, Huo D, Zhi S, Yang R, Yang B, et al. (2021). Astrocyte-Derived TGFβ1 Facilitates Blood-Brain Barrier Function via Non-Canonical Hedgehog Signaling in Brain Microvascular Endothelial Cells. Brain Sci, 11:77. 10.3390/brainsci11010077 PMC7826596 33430164

[56] Harding IC, O'Hare NR, Vigliotti M, Caraballo A, Lee CI, Millican K, et al. (2022). Developing a transwell millifluidic device for studying blood-brain barrier endothelium. Lab Chip, 22:4603-4620. 10.1039/d2lc00657j PMC11416711 36326069

[57] Hawkins RA, O'Kane RL, Simpson IA, Viña JR (2006). Structure of the blood-brain barrier and its role in the transport of amino acids. J Nutr, 136:218s-226s. 10.1093/jn/136.1.218S 16365086

[58] Cheng Y, Medina A, Yao Z, Basu M, Natekar JP, Lang J, et al. (2022). Intrinsic antiviral immunity of barrier cells revealed by an iPSC-derived blood-brain barrier cellular model. Cell Rep, 39:110885. 10.1016/j.celrep.2022.110885 PMC9230077 35649379

[59] Pan Y, Scanlon MJ, Owada Y, Yamamoto Y, Porter CJ, Nicolazzo JA (2015). Fatty Acid-Binding Protein 5 Facilitates the Blood-Brain Barrier Transport of Docosahexaenoic Acid. Mol Pharm, 12:4375-4385. 10.1021/acs.molpharmaceut.5b00580 26455443

[60] Beydoun R, Hamood MA, Gomez Zubieta DM, Kondapalli KC (2017). Na(+)/H(+) Exchanger 9 Regulates Iron Mobilization at the Blood-Brain Barrier in Response to Iron Starvation. J Biol Chem, 292:4293-4301. 10.1074/jbc.M116.769240 PMC5354498 28130443

[61] Richard C, Verdier F (2020). Transferrin Receptors in Erythropoiesis. Int J Mol Sci, 21:9713. 10.3390/ijms21249713 PMC7766611 33352721

[62] Koneru T, McCord E, Pawar S, Tatiparti K, Sau S, Iyer AK (2021). Transferrin: Biology and Use in Receptor-Targeted Nanotherapy of Gliomas. ACS Omega, 6:8727-8733. 10.1021/acsomega.0c05848 PMC8028004 33842744

[63] You L, Yu PP, Dong T, Guo W, Chang S, Zheng B, et al. (2022). Astrocyte-derived hepcidin controls iron traffic at the blood-brain-barrier via regulating ferroportin 1 of microvascular endothelial cells. Cell Death Dis, 13:667. 10.1038/s41419-022-05043-w PMC9343463 35915080

[64] Chang SL, Huang W, Mao X, Mack ML (2018). Ethanol's Effects on Transient Receptor Potential Channel Expression in Brain Microvascular Endothelial Cells. J Neuroimmune Pharmacol, 13:498-508. 10.1007/s11481-018-9796-3 PMC6431236 29987591

[65] Huang Z, Xu X, Li J, Gu L, Yue Y, Sun F, et al. (2022). RIG-I contributes to dsDNA-induced innate immune activation in human brain microvascular endothelial cells. Mol Immunol, 152:78-85. 10.1016/j.molimm.2022.10.009 36306644

[66] Rudolph H, Klopstein A, Gruber I, Blatti C, Lyck R, Engelhardt B (2016). Postarrest stalling rather than crawling favors CD8(+) over CD4(+) T-cell migration across the blood-brain barrier under flow in vitro. Eur J Immunol, 46:2187-2203. 10.1002/eji.201546251 PMC5113696 27338806

[67] Takata F, Nakagawa S, Matsumoto J, Dohgu S (2021). Blood-Brain Barrier Dysfunction Amplifies the Development of Neuroinflammation: Understanding of Cellular Events in Brain Microvascular Endothelial Cells for Prevention and Treatment of BBB Dysfunction. Front Cell Neurosci, 15:661838. 10.3389/fncel.2021.661838 PMC8475767 34588955

[68] Wimmer I, Tietz S, Nishihara H, Deutsch U, Sallusto F, Gosselet F, et al. (2019). PECAM-1 Stabilizes Blood-Brain Barrier Integrity and Favors Paracellular T-Cell Diapedesis Across the Blood-Brain Barrier During Neuroinflammation. Front Immunol, 10:711. 10.3389/fimmu.2019.00711 PMC6460670 31024547

[69] Marchetti L, Francisco D, Soldati S, Haghayegh Jahromi N, Barcos S, Gruber I, et al. (2022). ACKR1 favors transcellular over paracellular T-cell diapedesis across the blood-brain barrier in neuroinflammation in vitro. Eur J Immunol, 52:161-177. 10.1002/eji.202149238 PMC9293480 34524684

[70] van der Wijk AE, Lachkar N, de Vos J, Grootemaat AE, van der Wel NN, Hordijk PL, et al. (2019). Extravasation of Microspheres in a Rat Model of Silent Brain Infarcts. Stroke, 50:1590-1594. 10.1161/STROKEAHA.119.024975 31136287

[71] Gencer ES, Yilmaz E, Arsava EM, Gocmen R, Topcuoglu MA (2024). Cerebral arterial collateral status, but not venous outflow profiles, modifies the effect of intravenous tissue plasminogen activator in acute ischemic stroke. Brain Circ, 10:336-342. 10.4103/bc.bc_46_24 PMC11850934 40012595

[72] Rattner A, Williams J, Nathans J (2019). Roles of HIFs and VEGF in angiogenesis in the retina and brain. J Clin Invest, 129:3807-3820. 10.1172/JCI126655 PMC6715377 31403471

[73] Shi S, Tang M, Li H, Ding H, Lu Y, Gao L, et al. (2019). X-box binding protein l splicing attenuates brain microvascular endothelial cell damage induced by oxygen-glucose deprivation through the activation of phosphoinositide 3-kinase/protein kinase B, extracellular signal-regulated kinases, and hypoxia-inducible factor-1α/vascular endothelial growth factor signaling pathways. J Cell Physiol, 234:9316-9327. 10.1002/jcp.27614 30317635

[74] Wang C, Qu Y, Wang D, Zhu Y (2019). The Proangiogenic Roles of Long NonCoding RNAs Revealed by RNA-Sequencing Following Oxygen-Glucose Deprivation/Re-Oxygenation. Cell Physiol Biochem, 52:708-727. 10.33594/000000050 30921509

[75] Deng W, Fan C, Shen R, Wu Y, Du R, Teng J (2020). Long noncoding MIAT acting as a ceRNA to sponge microRNA-204-5p to participate in cerebral microvascular endothelial cell injury after cerebral ischemia through regulating HMGB1. J Cell Physiol, 235:4571-4586. 10.1002/jcp.29334 31628679

[76] Nishibori M, Wang D, Ousaka D, Wake H (2020). High Mobility Group Box-1 and Blood-Brain Barrier Disruption. Cells, 9:2650. 10.3390/cells9122650 PMC7764171 33321691

[77] Wu Z, Liang Y, Yu S (2020). Downregulation of microRNA-103a reduces microvascular endothelial cell injury in a rat model of cerebral ischemia by targeting AXIN2. J Cell Physiol, 235:4720-4733. 10.1002/jcp.29350 31650542

[78] Lee C, Chen R, Sun G, Liu X, Lin X, He C, et al. (2023). VEGF-B prevents excessive angiogenesis by inhibiting FGF2/FGFR1 pathway. Signal Transduct Target Ther, 8:305. 10.1038/s41392-023-01539-9 PMC10435562 37591843

[79] Anquetil T, Solinhac R, Jaffre A, Chicanne G, Viaud J, Darcourt J, et al. (2021). PI3KC2β inactivation stabilizes VE-cadherin junctions and preserves vascular integrity. EMBO Rep, 22:e51299. 10.15252/embr.202051299 PMC8183416 33880878

[80] Wang X, Dai Y, Zhang X, Pan K, Deng Y, Wang J, et al. (2021). CXCL6 regulates cell permeability, proliferation, and apoptosis after ischemia-reperfusion injury by modulating Sirt3 expression via AKT/FOXO3a activation. Cancer Biol Ther, 22:30-39. 10.1080/15384047.2020.1842705 PMC7834049 33241954

[81] Wendt TS, Gonzales RJ (2023). Ozanimod differentially preserves human cerebrovascular endothelial barrier proteins and attenuates matrix metalloproteinase-9 activity following in vitro acute ischemic injury. Am J Physiol Cell Physiol, 325:C951-C971. 10.1152/ajpcell.00342.2023 37642239

[82] Diaz-Cañestro C, Merlini M, Bonetti NR, Liberale L, Wüst P, Briand-Schumacher S, et al. (2018). Sirtuin 5 as a novel target to blunt blood-brain barrier damage induced by cerebral ischemia/reperfusion injury. Int J Cardiol, 260:148-155. 10.1016/j.ijcard.2017.12.060 29622432

[83] Burek M, König A, Lang M, Fiedler J, Oerter S, Roewer N, et al. (2019). Hypoxia-Induced MicroRNA-212/132 Alter Blood-Brain Barrier Integrity Through Inhibition of Tight Junction-Associated Proteins in Human and Mouse Brain Microvascular Endothelial Cells. Transl Stroke Res, 10:672-683. 10.1007/s12975-018-0683-2 PMC6842347 30617994

[84] Gong S, Cao G, Li F, Chen Z, Pan X, Ma H, et al. (2021). Endothelial Conditional Knockdown of NMMHC IIA (Nonmuscle Myosin Heavy Chain IIA) Attenuates Blood-Brain Barrier Damage During Ischemia-Reperfusion Injury. Stroke, 52:1053-1064. 10.1161/STROKEAHA.120.031410 33588591

[85] Chen J, Sun L, Ding GB, Chen L, Jiang L, Wang J, et al. (2019). Oxygen-Glucose Deprivation/Reoxygenation Induces Human Brain Microvascular Endothelial Cell Hyperpermeability Via VE-Cadherin Internalization: Roles of RhoA/ROCK2. J Mol Neurosci, 69:49-59. 10.1007/s12031-019-01326-8 31187440

[86] Zhu J, Li Z, Ji Z, Wu Y, He Y, Liu K, et al. (2022). Glycocalyx is critical for blood-brain barrier integrity by suppressing caveolin1-dependent endothelial transcytosis following ischemic stroke. Brain Pathol, 32:e13006. 10.1111/bpa.13006 PMC8713524 34286899

[87] Chen P, Zhang H, Zhang Q, Zhou W, Deng Y, Hu X, et al. (2019). Basic Fibroblast Growth Factor Reduces Permeability and Apoptosis of Human Brain Microvascular Endothelial Cells in Response to Oxygen and Glucose Deprivation Followed by Reoxygenation via the Fibroblast Growth Factor Receptor 1 (FGFR1)/ERK Pathway. Med Sci Monit, 25:7191-7201. 10.12659/MSM.918626 PMC6778414 31551405

[88] Yan G, Zhao H, Hong X (2020). LncRNA MACC1-AS1 attenuates microvascular endothelial cell injury and promotes angiogenesis under hypoxic conditions via modulating miR-6867-5p/TWIST1 in human brain microvascular endothelial cells. Ann Transl Med, 8:876. 10.21037/atm-20-4915 PMC7396759 32793720

[89] Yan Y, Wang Z, Liu X, Han S, Li J, Zhang Y, et al. (2023). Identification of brain endothelial cell-specific genes and pathways in ischemic stroke by integrated bioinformatical analysis. Brain Circ, 9:228-239. 10.4103/bc.bc_40_23 PMC10821689 38284111

[90] Seo JH, Maki T, Miyamoto N, Choi YK, Chung KK, Hamanaka G, et al. (2020). AKAP12 Supports Blood-Brain Barrier Integrity against Ischemic Stroke. Int J Mol Sci, 21:9078. 10.3390/ijms21239078 PMC7730430 33260683

[91] Yang J, Wang Z, Liu X, Lu P (2021). Modulation of vascular integrity and neuroinflammation by peroxiredoxin 4 following cerebral ischemia-reperfusion injury. Microvasc Res, 135:104144. 10.1016/j.mvr.2021.104144 33515567

[92] Yang Z, Lin P, Chen B, Zhang X, Xiao W, Wu S, et al. (2021). Autophagy alleviates hypoxia-induced blood-brain barrier injury via regulation of CLDN5 (claudin 5). Autophagy, 17:3048-3067. 10.1080/15548627.2020.1851897 PMC8526012 33280500

[93] Li Z, Li J, Tang N (2017). Long noncoding RNA Malat1 is a potent autophagy inducer protecting brain microvascular endothelial cells against oxygen-glucose deprivation/reoxygenation-induced injury by sponging miR-26b and upregulating ULK2 expression. Neuroscience, 354:1-10. 10.1016/j.neuroscience.2017.04.017 28433650

[94] Wang S, Han X, Mao Z, Xin Y, Maharjan S, Zhang B (2019). MALAT1 lncRNA Induces Autophagy and Protects Brain Microvascular Endothelial Cells Against Oxygen-Glucose Deprivation by Binding to miR-200c-3p and Upregulating SIRT1 Expression. Neuroscience, 397:116-126. 10.1016/j.neuroscience.2018.11.024 30496821

[95] Peng Z, Ji D, Qiao L, Chen Y, Huang H (2021). Autophagy Inhibition by ATG3 Knockdown Remits Oxygen-Glucose Deprivation/Reoxygenation-Induced Injury and Inflammation in Brain Microvascular Endothelial Cells. Neurochem Res, 46:3200-3212. 10.1007/s11064-021-03423-w 34379294

[96] Spitzer D, Guérit S, Puetz T, Khel MI, Armbrust M, Dunst M, et al. (2022). Profiling the neurovascular unit unveils detrimental effects of osteopontin on the blood-brain barrier in acute ischemic stroke. Acta Neuropathol, 144:305-337. 10.1007/s00401-022-02452-1 PMC9288377 35752654

[97] Shekhar S, Liu Y, Wang S, Zhang H, Fang X, Zhang J, et al. (2021). Novel Mechanistic Insights and Potential Therapeutic Impact of TRPC6 in Neurovascular Coupling and Ischemic Stroke. Int J Mol Sci, 22:2074. 10.3390/ijms22042074 PMC7922996 33669830

[98] Li W, Mandeville ET, Durán-Laforet V, Fukuda N, Yu Z, Zheng Y, et al. (2022). Endothelial cells regulate astrocyte to neural progenitor cell trans-differentiation in a mouse model of stroke. Nat Commun, 13:7812. 10.1038/s41467-022-35498-6 PMC9763251 36535938

[99] Ozaki T, Nakamura H, Kishima H (2019). Therapeutic strategy against ischemic stroke with the concept of neurovascular unit. Neurochem Int, 126:246-251. 10.1016/j.neuint.2019.03.022 30946849

[100] Liu Z, Chopp M (2016). Astrocytes, therapeutic targets for neuroprotection and neurorestoration in ischemic stroke. Prog Neurobiol, 144:103-120. 10.1016/j.pneurobio.2015.09.008 PMC4826643 26455456

[101] Liu J, Li J (2022). Astrocytes Protect Human Brain Microvascular Endothelial Cells from Hypoxia Injury by Regulating VEGF Expression. J Healthc Eng, 2022:1884959. 10.1155/2022/1884959 PMC8956445 35340230

[102] Wu KW, Lv LL, Lei Y, Qian C, Sun FY (2019). Endothelial cells promote excitatory synaptogenesis and improve ischemia-induced motor deficits in neonatal mice. Neurobiol Dis, 121:230-239. 10.1016/j.nbd.2018.10.006 30308244

[103] Kou ZW, Mo JL, Wu KW, Qiu MH, Huang YL, Tao F, et al. (2019). Vascular endothelial growth factor increases the function of calcium-impermeable AMPA receptor GluA2 subunit in astrocytes via activation of protein kinase C signaling pathway. Glia, 67:1344-1358. 10.1002/glia.23609 PMC6594043 30883902

[104] Michinaga S, Koyama Y (2019). Dual Roles of Astrocyte-Derived Factors in Regulation of Blood-Brain Barrier Function after Brain Damage. Int J Mol Sci, 20:571. 10.3390/ijms20030571 PMC6387062 30699952

[105] Espinoza-Rojo M, Iturralde-Rodríguez KI, Chánez-Cárdenas ME, Ruiz-Tachiquín ME, Aguilera P (2010). Glucose transporters regulation on ischemic brain: possible role as therapeutic target. Cent Nerv Syst Agents Med Chem, 10:317-325. 10.2174/187152410793429755 20868355

[106] Hong J, Kim Y, Yanpallewar S, Lin PC (2020). The Rho/Rac Guanine Nucleotide Exchange Factor Vav1 Regulates Hif-1α and Glut-1 Expression and Glucose Uptake in the Brain. Int J Mol Sci, 21:1341. 10.3390/ijms21041341 PMC7072975 32079227

[107] Yuen N, Lam TI, Wallace BK, Klug NR, Anderson SE, O'Donnell ME (2014). Ischemic factor-induced increases in cerebral microvascular endothelial cell Na/H exchange activity and abundance: evidence for involvement of ERK1/2 MAP kinase. Am J Physiol Cell Physiol, 306:C931-942. 10.1152/ajpcell.00021.2013 PMC4024711 24647544

[108] Wang P, Pan R, Weaver J, Jia M, Yang X, Yang T, et al. (2021). MicroRNA-30a regulates acute cerebral ischemia-induced blood-brain barrier damage through ZnT4/zinc pathway. J Cereb Blood Flow Metab, 41:641-655. 10.1177/0271678X20926787 PMC7922758 32501158

[109] DeMars KM, Yang C, Hawkins KE, McCrea AO, Siwarski DM, Candelario-Jalil E (2017). Spatiotemporal Changes in P-glycoprotein Levels in Brain and Peripheral Tissues Following Ischemic Stroke in Rats. J Exp Neurosci, 11:1179069517701741. 10.1177/1179069517701741 PMC5398227 28469478

[110] Wang F, Ji S, Wang M, Liu L, Li Q, Jiang F, et al. (2020). HMGB1 promoted P-glycoprotein at the blood-brain barrier in MCAO rats via TLR4/NF-κB signaling pathway. Eur J Pharmacol, 880:173189. 10.1016/j.ejphar.2020.173189 32417325

[111] Huang L, Chen Y, Liu R, Li B, Fei X, Li X, et al. (2022). P-Glycoprotein Aggravates Blood Brain Barrier Dysfunction in Experimental Ischemic Stroke by Inhibiting Endothelial Autophagy. Aging Dis, 13:1546-1561. 10.14336/AD.2022.0225 PMC9466967 36186136

[112] Hernandes MS, Xu Q, Griendling KK (2022). Role of NADPH Oxidases in Blood-Brain Barrier Disruption and Ischemic Stroke. Antioxidants (Basel), 11:1966. 10.3390/antiox11101966 PMC9598888 36290688

[113] Kim Y, Lee S, Zhang H, Lee S, Kim H, Kim Y, et al. (2020). CLEC14A deficiency exacerbates neuronal loss by increasing blood-brain barrier permeability and inflammation. J Neuroinflammation, 17:48. 10.1186/s12974-020-1727-6 PMC7001304 32019570

[114] Bellut M, Papp L, Bieber M, Kraft P, Stoll G, Schuhmann MK (2021). NLPR3 inflammasome inhibition alleviates hypoxic endothelial cell death in vitro and protects blood-brain barrier integrity in murine stroke. Cell Death Dis, 13:20. 10.1038/s41419-021-04379-z PMC8688414 34930895

[115] Huang Y, Chen S, Luo Y, Han Z (2020). Crosstalk between Inflammation and the BBB in Stroke. Curr Neuropharmacol, 18:1227-1236. 10.2174/1570159X18666200620230321 PMC7770647 32562523

[116] Winneberger J, Schöls S, Lessmann K, Rández-Garbayo J, Bauer AT, Mohamud Yusuf A, et al. (2021). Platelet endothelial cell adhesion molecule-1 is a gatekeeper of neutrophil transendothelial migration in ischemic stroke. Brain Behav Immun, 93:277-287. 10.1016/j.bbi.2020.12.026 33388423

[117] Bernstein DL, Zuluaga-Ramirez V, Gajghate S, Reichenbach NL, Polyak B, Persidsky Y, et al. (2020). miR-98 reduces endothelial dysfunction by protecting blood-brain barrier (BBB) and improves neurological outcomes in mouse ischemia/reperfusion stroke model. J Cereb Blood Flow Metab, 40:1953-1965. 10.1177/0271678X19882264 PMC7786850 31601141

[118] Candelario-Jalil E, Dijkhuizen RM, Magnus T (2022). Neuroinflammation, Stroke, Blood-Brain Barrier Dysfunction, and Imaging Modalities. Stroke, 53:1473-1486. 10.1161/STROKEAHA.122.036946 PMC9038693 35387495

[119] Zhang L, Wei W, Ai X, Kilic E, Hermann DM, Venkataramani V, et al. (2021). Extracellular vesicles from hypoxia-preconditioned microglia promote angiogenesis and repress apoptosis in stroke mice via the TGF-β/Smad2/3 pathway. Cell Death Dis, 12:1068. 10.1038/s41419-021-04363-7 PMC8578653 34753919

[120] Zhang L, Graf I, Kuang Y, Zheng X, Haupt M, Majid A, et al. (2021). Neural Progenitor Cell-Derived Extracellular Vesicles Enhance Blood-Brain Barrier Integrity by NF-κB (Nuclear Factor-κB)-Dependent Regulation of ABCB1 (ATP-Binding Cassette Transporter B1) in Stroke Mice. Arterioscler Thromb Vasc Biol, 41:1127-1145. 10.1161/ATVBAHA.120.315031 PMC7901534 33327747

[121] Li H, Liu P, Zhang B, Yuan Z, Guo M, Zou X, et al. (2023). Acute ischemia induces spatially and transcriptionally distinct microglial subclusters. Genome Med, 15:109. 10.1186/s13073-023-01257-5 PMC10712107 38082331

[122] Alwjwaj M, Kadir RRA, Bayraktutan U (2022). Outgrowth endothelial progenitor cells restore cerebral barrier function following ischaemic damage: The impact of NOX2 inhibition. Eur J Neurosci, 55:1658-1670. 10.1111/ejn.15627 35179812

[123] Phelps J, Hart DA, Mitha AP, Duncan NA, Sen A (2023). Physiological oxygen conditions enhance the angiogenic properties of extracellular vesicles from human mesenchymal stem cells. Stem Cell Res Ther, 14:218. 10.1186/s13287-023-03439-9 PMC10463845 37612731

[124] Wang J, Chen Y, Yang Y, Xiao X, Chen S, Zhang C, et al. (2016). Endothelial progenitor cells and neural progenitor cells synergistically protect cerebral endothelial cells from Hypoxia/reoxygenation-induced injury via activating the PI3K/Akt pathway. Mol Brain, 9:12. 10.1186/s13041-016-0193-7 PMC4738765 26842559

[125] Li S, Hafeez A, Noorulla F, Geng X, Shao G, Ren C, et al. (2017). Preconditioning in neuroprotection: From hypoxia to ischemia. Prog Neurobiol, 157:79-91. 10.1016/j.pneurobio.2017.01.001 PMC5515698 28110083

[126] Liu J, Gu Y, Guo M, Ji X (2021). Neuroprotective effects and mechanisms of ischemic/hypoxic preconditioning on neurological diseases. CNS Neurosci Ther, 27:869-882. 10.1111/cns.13642 PMC8265941 34237192

[127] Zhao M, Qiao Y, Weiss A, Zhao W (2024). Neuroprotective strategies in acute ischemic stroke: A narrative review of recent advances and clinical outcomes. Brain Circ, 10:296-302. 10.4103/bc.bc_165_24 PMC11850939 40012592

[128] Xu Y, Wang Y, Ji X (2023). Immune and inflammatory mechanism of remote ischemic conditioning: A narrative review. Brain Circ, 9:77-87. 10.4103/bc.bc_57_22 PMC10419737 37576576

[129] Gu T, Just J, Stenz KT, Yan Y, Sieljacks P, Wang J, et al. (2022). The Role of Plasma Extracellular Vesicles in Remote Ischemic Conditioning and Exercise-Induced Ischemic Tolerance. Int J Mol Sci, 23:3334. 10.3390/ijms23063334 PMC8951333 35328755

[130] Ren C, Li S, Wang B, Han R, Li N, Gao J, et al. (2018). Limb remote ischemic conditioning increases Notch signaling activity and promotes arteriogenesis in the ischemic rat brain. Behav Brain Res, 340:87-93. 10.1016/j.bbr.2016.10.036 27780723

[131] Liang D, He XB, Wang Z, Li C, Gao BY, Wu JF, et al. (2018). Remote limb ischemic postconditioning promotes motor function recovery in a rat model of ischemic stroke via the up-regulation of endogenous tissue kallikrein. CNS Neurosci Ther, 24:519-527. 10.1111/cns.12813 PMC6489769 29399973

[132] Saito M, Hoshino T, Ishizuka K, Iwasaki S, Toi S, Shibata N, et al. (2024). Remote Ischemic Conditioning Enhances Collateral Circulation Through Leptomeningeal Anastomosis and Diminishes Early Ischemic Lesions and Infarct Volume in Middle Cerebral Artery Occlusion. Transl Stroke Res, 15:41-52. 10.1007/s12975-022-01108-2 36441491

[133] Ma Y, Sun W, Bai J, Gao F, Ma H, Liu H, et al. (2024). Targeting blood brain barrier-Remote ischemic conditioning alleviates cognitive impairment in female APP/PS1 rats. CNS Neurosci Ther, 30:e14613. 10.1111/cns.14613 PMC10879645 38379185

[134] He Q, Ma Y, Fang C, Deng Z, Wang F, Qu Y, et al. (2023). Remote ischemic conditioning attenuates blood-brain barrier disruption after recombinant tissue plasminogen activator treatment via reducing PDGF-CC. Pharmacol Res, 187:106641. 10.1016/j.phrs.2022.106641 36587812

[135] Wang Q, Kohls W, Wills M, Li F, Pang Q, Geng X, et al. (2024). A novel stroke rehabilitation strategy and underlying stress granule regulations through inhibition of NLRP3 inflammasome activation. CNS Neurosci Ther, 30:e14405. 10.1111/cns.14405 PMC10805392 37580991

[136] Geng X, Wang Q, Lee H, Huber C, Wills M, Elkin K, et al. (2021). Remote Ischemic Postconditioning vs. Physical Exercise After Stroke: an Alternative Rehabilitation Strategy^+^ Mol Neurobiol, 58:3141-3157. 10.1007/s12035-021-02329-6 PMC8257517 33625674

[137] Ren C, Li N, Li S, Han R, Huang Q, Hu J, et al. (2018). Limb Ischemic Conditioning Improved Cognitive Deficits via eNOS-Dependent Augmentation of Angiogenesis after Chronic Cerebral Hypoperfusion in Rats. Aging Dis, 9:869-879. 10.14336/AD.2017.1106 PMC6147592 30271664

[138] Khan MB, Hafez S, Hoda MN, Baban B, Wagner J, Awad ME, et al. (2018). Chronic Remote Ischemic Conditioning Is Cerebroprotective and Induces Vascular Remodeling in a VCID Model. Transl Stroke Res, 9:51-63. 10.1007/s12975-017-0555-1 PMC5750336 28755277

[139] Baillieul S, Chacaroun S, Doutreleau S, Detante O, Pépin JL, Verges S (2017). Hypoxic conditioning and the central nervous system: A new therapeutic opportunity for brain and spinal cord injuries^+^ Exp Biol Med (Maywood), 242:1198-1206. 10.1177/1535370217712691 PMC5478009 28585890

[140] Ou K, Mertsch S, Theodoropoulou S, Wu J, Liu J, Copland DA, et al. (2019). Müller Cells Stabilize Microvasculature through Hypoxic Preconditioning. Cell Physiol Biochem, 52:668-680. 10.33594/000000047 30921506

[141] Guan Y, Gu Y, Shao H, Ma W, Li G, Guo M, et al. (2023). Intermittent hypoxia protects against hypoxic-ischemic brain damage by inducing functional angiogenesis. J Cereb Blood Flow Metab, 43:1656-1671. 10.1177/0271678X231185507 PMC10581229 37395346

[142] Jiang RH, Wu CJ, Xu XQ, Lu SS, Zu QQ, Zhao LB, et al. (2019). Hypoxic conditioned medium derived from bone marrow mesenchymal stromal cells protects against ischemic stroke in rats. J Cell Physiol, 234:1354-1368. 10.1002/jcp.26931 30076722

[143] Stowe AM, Altay T, Freie AB, Gidday JM (2011). Repetitive hypoxia extends endogenous neurovascular protection for stroke. Ann Neurol, 69:975-985. 10.1002/ana.22367 PMC3117913 21437933

